# Improved facial emotion recognition model based on a novel deep convolutional structure

**DOI:** 10.1038/s41598-024-79167-8

**Published:** 2024-11-23

**Authors:** Reham A. Elsheikh, M. A. Mohamed, Ahmed Mohamed Abou-Taleb, Mohamed Maher Ata

**Affiliations:** 1 Department of Electronics and Communications Engineering, Faculty of Engineering, Mansoura University, Mansoura, Egypt; 2 School of Computational Sciences and Artificial Intelligence (CSAI), Zewail City of Science and Technology, October Gardens, 6th of October City, 12578 Giza Egypt

**Keywords:** Anti-aliasing, Emotion recognition, Deep learning, Convolutional neural network, Facial recognition, Psychology, Engineering

## Abstract

Facial Emotion Recognition (FER) is a very challenging task due to the varying nature of facial expressions, occlusions, illumination, pose variations, cultural and gender differences, and many other aspects that cause a drastic degradation in quality of facial images. In this paper, an anti-aliased deep convolution network (AA-DCN) model has been developed and proposed to explore how anti-aliasing can increase and improve recognition fidelity of facial emotions. The AA-DCN model detects eight distinct emotions from image data. Furthermore, their features have been extracted using the proposed model and numerous classical deep learning algorithms. The proposed AA-DCN model has been applied to three different datasets to evaluate its performance: The Cohn-Kanade Extending (CK+) database has been utilized, achieving an ultimate accuracy of 99.26% in (5 min, 25 s), the Japanese female facial expressions (JAFFE) obtained 98% accuracy in (8 min, 13 s), and on one of the most challenging FER datasets; the Real-world Affective Face (RAF) dataset; reached 82%, in low training time (12 min, 2s). The experimental results demonstrate that the anti-aliased DCN model is significantly increasing emotion recognition while improving the aliasing artifacts caused by the down-sampling layers.

## Introduction

In human communication, emotions are the first signs to express how they feel on the inside. These emotions enable them to communicate with one another, with their environment, and that has been revolutionizing the way they interact with technology: either through their facial expressions, physiological signals, or tone of voice^1^. In daily life, the influence of facial expressions on overall communication varies from 55 to 93%. So, a large amount of useful emotional data may be acquired by detecting facial expressions^2^. This is why, when compared to other technologies, automated FER has received the greatest attention from researchers. Automated FER has been widely applied in the discipline of computer vision such as human-computer-interactions, smartphones, security, behavioral psychology (criminal psychic analysis), medical treatment, observation of driver exhaustion, animation, and other fields^3^. It is also a fundamental technique in robot vision, allowing robots to understand human emotions. For many years, Deep Convolutional Neural Networks (DCNs) had been considered to be invariant to low image transformations such as scaling, image translation, and other minor modifications. As a result, they are frequently employed in the recognition of facial emotions. However, numerous researchers have lately demonstrated that this is not the case and that DCNs are truly shift-variants^4^. One frequent reason is down-sampling (stride) strategies that disregard the sampling theorem, yielding in the aliasing problem. Aliasing in DCN happens when high-frequency image components are mistakenly represented as low-frequency ones during the down-sampling process, leading to data loss. This causes a loss of critical features and jagged edges, which can negatively impact the DCN’s overall performance. For example, aliasing might allow DCN to incorrectly label one emotion with another when performing facial emotion classification tasks, leading to a significant decrease in accuracy. Anti-aliasing is one potential fix to this issue, which employs a significant signal processing principle, which is one ought to always blur just before subsampling, yet recent CNN architectures do not follow this approach^5^. Unlike numerous prior studies that employed antialiasing techniques in deep learning (DL), this work presented antialiasing in a CNN methodology designed to tackle the aliasing difficulty in FER systems.

For example, Zou et al.^6^ developed an enhanced low-pass filtration layer that addresses aliasing issues, which is an obstacle in deep learning. This layer functions to estimate filter weights for each channel group and spatial location in the input feature maps. The approach was then evaluated on a variety of applications, including COCO instance segmentation, ImageNet categorization, and segment landscapes. The results indicate that this technique easily responds to different feature frequencies, eliminating aliasing while retaining key identifying information^7^. Furthermore, Ning et al. recently employed the currently available WaveCNet anti-aliasing approach for tiny-object identification. In each ResNet residual block pathway, the authors deployed WaveletPool uniformly. WaveCNet reliably avoids aliasing by replacing standard down-sampling procedures in CNNs with wavelet pooling (WaveletPool) layers. Experiments on the WiderFace, DOTA, and TinyPerson datasets demonstrate how important anti-aliasing is for tiny object detection and how competently the recommended method succeeds in yielding new state-of-the-art results on all three datasets^8^.

**Fig. 1:**
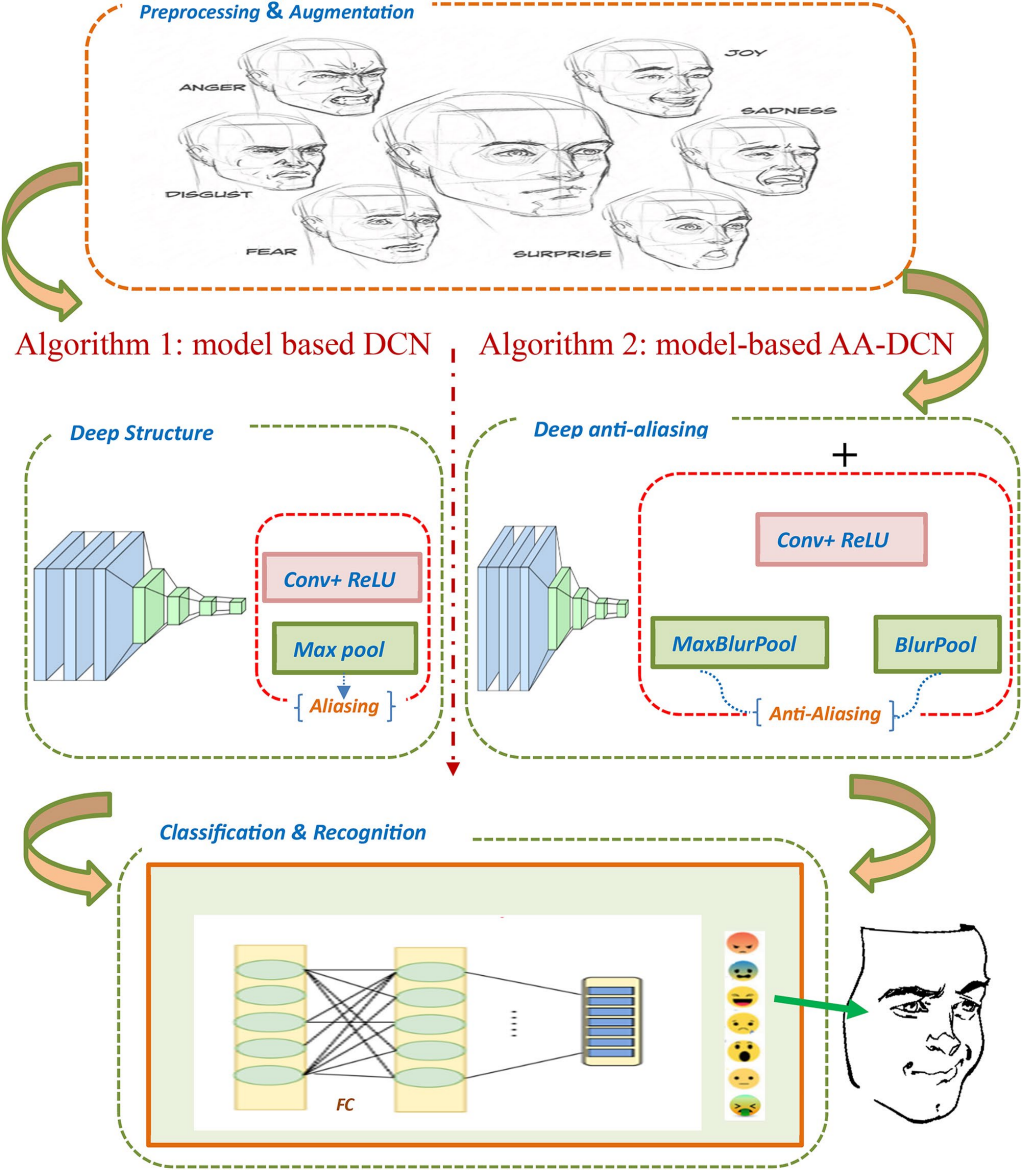
The proposed model’s fundamental architecture.

In this paper, after the dataset collection, preprocessing, augmentation, and by analyzing the recognition accuracy of traditional CNN models, the datasets go through two main phases: (i) The first phase has been adopted to extract features from facial images based on an optimized deep CNN model. (iii) The second phase has been employed based on a hybrid (AA-DCN) model using a tuned blur filter to achieve an optimal anti-aliasing effect, resulting in a more accurate emotion recognition. Figure 1 shows the main components of the two proposed recognition algorithms.

The key contributions made in this research can be summarized as follows:

- Preprocessing and augmenting the utilized datasets to expand and balance their size for enhancing the training capacity.
- Evaluating and analyzing the performance of some classical (VGG16, VGG19, InceptionV3, Xception, EfficientNetB0, ResNet50, and DenseNet121) deep CNN models for classifying emotions from facial expressions.
- Proposing a deep learning-based DCN approach (Algorithm 1) to extract features that provide a magnificent impact for enhanced facial emotion recognition.
- Proposing a hybrid (AA-DCN) model (Algorithm 2) using a tuned blur-pool layers and max-pool layers in the DCN model to increase accuracy of emotion recognition by increasing quality of images for an optimal anti-aliasing effect.
- Modifying the hyper-parameters of both proposed models while testing the suggested models on various FER benchmark datasets.
- Comparing performance of existing studies with the proposed approaches.

The following work is organized as follows: Sect. (2) discusses related work, Sect. (3) focuses on classical CNN architectures, Sect. (4) introduces the developed DCN method employed in this study, Sect. (5) describes the utilized datasets, Sect. (6) explores the experimental outcomes and discussions of the applied DCN methodology, Sect. (7) presents the AA-DCN as well as the detailed experimental process, and Sect. (8) draws a conclusion of the paper, discusses the limits in this study and makes suggestions for further developments.

## Related work

A novel FER system has been presented by Umer et al. using deep learning. The algorithm has been divided into four steps: (a) a face detection process to define a region of interest, (b) feature learning tasks through a CNN architecture, and (c) techniques for data augmentation have been employed to fertilize the learning that leads to a great enhancement in the performance of the FER-method. For that, the experimental results showed high performance in comparison to current state-of-the-art approaches^3^. Chowdary et al. have investigated the transfer learning approaches for facial emotion classification. The authors have eliminated only the fully connected layers of the pre-trained models and added new fully-connected layers that were more suitable for the instructions of the task. The mobile-Net model achieved the highest performance among all four pre-trained models because of its faster performance, and a small number of parameters. One of the limitations of the proposed model was using only one dataset in testing the experiments^9^. Abate et al. have investigated the influence of masked faces on recognizing emotions from facial images. They have discussed how the most performing algorithms like CNN, ResNet, and ARM could be retrained in three different occlusion scenarios in the presence of facial masks. The results reported in this study were useful to draw attention to the challenging occlusion problem, but they were not the best^10^. Shaik et al. have aimed to develop a novel deep learning strategy known as the “Visual-Attention-based Composite Dense Neural Network” (VA-CDNN) that focuses on extracting attention-based features from several faces. Therefore, to extract global features from a normalized face, Viola-Jones methods and the Xception model have been used to extract localized landmarks (the mouth and eye pairs). Then, to categorize the facial expressions, a deep neural network has been constructed that accepts both local and global attention information. Even the suggested model outperformed many recent advances in FER, but this strategy only operated on frontal pictures and was confined to real-time invariant face data^11^. Saurav et al. have published a real-time Dual Integrated-CNN (DICNN) model for facial emotion categorization in the wild. Face detection, alignment and recognition using the suggested DICNN model are the three phases of the FER approach. This methodology was developed and implemented on an embedded-platform. Although the model has efficiently recognized facial expressions, it faces poor misclassification, mostly in the fear category^12^.

Rajan et al., on the other hand, have presented a hybrid, layered CNN methodology for real-time FER. The model proposed is split up into three stages: First, two pre-processing procedures have been conducted, one to improve the edges and another to cope with illumination variations. Second, weighted histogram equalization (input1) and edge enhancement (input2) have been fed into a dual CNN layer for the feature maps. Finally, these characteristics have been integrated and included in the LSTM. They have been then connected to the global average pooling (GAP) to reduce the characteristics. Following that, the SoftMax layer estimated the expression. This model has been evaluated using a self-created database as well as three publicly available FER datasets. The recommended approach performs well in distinguishing surprised and joyful reactions but badly in sadness and anger^13^. Khattak et al. have revealed a CNN technique for classifying age, emotions, and gender from face data. Unlike the prior studies, which faced a problem of degradation in image quality resulted from the mis-selection of CNN layers, this model utilized an appropriately optimized number of layers to improve the classification accuracy. However, the experiments carried out on gender and age employed just one domain dataset, and the other datasets used in classification were restricted^14^.

Bentomi et al. have presented a hybrid approach for FER associating (VGG16, ResNet50) with a multilayer perceptron (MLP) classifier. The classical models have been employed as feature extractors by adding only the GAP layer; no fine-tuning was done to the network parameters. The early-stopping has been utilized to avert overfitting in MLP and has also improved the overall accuracy in terms of generalization. The method still needs to be tested on large datasets for recognizing facial emotions^15^. LIU et al. have performed a new deep-learning model to improve the prediction accuracy from face emotion. To combat the effects of ambient noise, a pose-guided face alignment approach has been developed to eliminate intra-class differences. A fusion ResNet and VGG-16 model has also been created to reduce training time. The suggested approach has various benefits, including the complete utilization of facial alignment to minimize the influence of ambient noise, including changes in posture, lighting, and occlusion. Furthermore, the model efficiently distinguishes between comparable sentiments such as fear and disgust. However, the classification performance still has to be improved^16^.

Wang et al. have coupled the benefits of the attention mechanism with multi-task learning. The suggested multi-task attention network (MTAN) has been enhanced in two ways: task and feature. Using the self-attention mechanism, the MTACN network focused on the relevance of each attention module for each unique activity. Furthermore, the MTCAN model has been presented to solve the problem of task divergence. As a result, the self-attention mechanism is added to capture the distance dependency between the attention modules of particular tasks, depending on the two tasks (classification and regression). The aspects of each activity are then thoroughly learned. The suggested classification task and emotion recognition accuracy still need to be improved^17^. Taskiran et al. have proposed another hybrid face recognition (HFR) method to increase the robustness of face recognition. The HFR system is comprised of six steps: face detection from video frames; detection of facial landmarks to extract dynamic characteristics during smiling action; extraction of appearance features from landmarks during a smile using 3 different pretrained architectures (ArcFace, VGGFace, and VGGFace2); extraction of dynamic facial features for gender detection; and feature selection and classification using an Extremely-Randomized-Trees Classifier. The proposed model could be useful for performing face recognition in videos extracted from systems that may contain images with illumination variations, noise, and blur while performing face recognition. However, the accuracy of performing face recognition needs to be improved for better face recognition^18^. EmNet (Emotion Network), a deep integrated CNN model, has been investigated by Saurav et al. The EmNet model improved the integrated variation of two structurally comparable Deep-CNN models using a joint optimization approach. The new FER technique’s efficiency has been evaluated on an embedded platform with limited resources, and it achieved a significant gain in accuracy over current methods. Furthermore, EmNet’s three prediction outputs were joined using two integration algorithms (averaged and weighted maximum). The suggested model functioned well in identifying facial images in the neutral, surprise, disgust, and happiness classes but struggled in the sad and afraid classes^19^.

Devi D et al. have used a novel Deep Regression (DR) classifier to recognize facial emotions. The DR model is divided into six phases: pre-processing with the Gamma-HE algorithm, facial point extraction with the Pyramid Histogram of Oriented-Gradients (PHOG) algorithm, segmentation with the Viola-Jones Algorithm (VJA), feature extraction, feature selection, and finally classification. In comparison to current algorithms, the presented FER model earned significant accuracy. However, the major issue in this work was the high training time^20^. Li et al. have presented an improved FER methodology based ResNet-50. The method uses a CNN model for expression recognition. Also, to overcome the overfitting problem that may occur, the 10-group cross-validation technique has been chosen. Each group consisted of 10 images representing the seven emotions. Even though the proposed technique had a good recognition effect and good accuracy, more images were needed to be collected than in this experiment to make further improvements in facial recognition^21^. Arora et al. have presented a hybrid automatic system that could differentiate the emotions connoted on the face. Principal Component Analysis (PCA) and a gradient filter were obtained for feature extraction, and Particle Swarm Optimization (PSO) is used to optimize the extracted features for each emotion.

The authors have achieved high classification accuracy, but with only one dataset in the testing phase^22^. Zheng et al. have constructed a hybrid Inception ResNetV2 and attention mechanism called Convolutional Block Attention Module (CBAM) to increase the capacity of instructors to recognize expressions in real-world environments. The Inception ResNet V2 was utilized to extract the deep expression features and was deployed as a globalization network to mitigate the issue of over-fitting during the learning phase. The attention module (CBAM) is included to focus on the expression details. In addition, a new dataset of intensity-based facial expressions known as EIDB-13 is generated. The model might also assess students’ interest in educational material. For better feature extraction, this method needs to be optimized further^23^.

Fontaine et al. have focused their research on the role of AI in assessing postoperative pain. To categorize and identify distinct patients’ facial expressions, a DCNN system (ResNet-18) is presented and evaluated. Their data has been collected before to and following surgery using self-reported pain intensity (NRS, from 0 to 10). The suggested DL method accurately predicted pain intensity among these 11 available ratings. The findings indicated that facial expression analysis-based AI might be highly beneficial in recognizing severe pain, particularly in persons who are unable to adequately describe their suffering. However, the scientists did not compare the expected results to human observers’ assessments. They were also utilizing a pre-trained ResNet-18 model due to the low data availability^24^. Ching et al. have presented a real-time entertainment greeting system using the CNN model to improve the down mood of any passersby. The CNN model has been used to detect eyes, faces, and mouths from a captured image using the VJA. The emotions are recognized from the eyes and mouth, where the face is used to recognize a known user. After that, a funny 3-D animation is played depending on the specified mood. The experimental results showed that the presented model recognized and identified the face and emotion well. But the proposed approach was limited to three emotions only (happy, regular, and unhappy), as the main aim of the system was to locate passers-by who were unhappy^25^.

A deep convolution neural network approach based on a local gravitational force descriptor was presented by Mohan et al. as a means of classifying FERs. There are two components to the suggested approach. A unique deep convolution neural network model (DCNN) is fed with the local gravitational force descriptor, which was first used to extract local characteristics from face photos. Two branches make up the provided DCNN. While the second branch extracts holistic information, the first branch was used to identify geometric aspects, including edges, curves, and lines. Lastly, the final categorization score is calculated using the score-level fusion approach. The long training time of this work hindered its performance, even if the findings show that it beat all state-of-the-art approaches on all databases^6^. Furthermore, FER-net—a convolutional neural network designed to effectively differentiate FEs—was developed by Mohan et al. Features are automatically extracted from facial regions using FER-net. After that, a Softmax classifier received these features in order to identify FEs. FER-net was evaluated on five benchmarking datasets: FER2013, Jaffee, CK+, KDEF, and RAF. These datasets have average accuracy rates of 78.9%, 96.7%, 97.8%, 82.5%, and 81.68%, respectively. The acquired findings show that the FER-net is superior when compared to recent research^26^.

A deep convolutional neural network called LieNet was developed by Mohan et al. to accurately and identify the multiscale variations of deceit. To create a single image, the first 20 frames from each movie are retrieved and synthesized. Additionally, a signal with audio is taken out of the video. In addition, a 2D plane is plotted with 13 channels of EEG signals, and these signals are concatenated to create a image. Second, features were taken out of each modality independently by the LieNet model. Third, a Softmax classifier is used to estimate scores across all modalities. Experimental results show that the LieNet outperforms previous research on the BoL database’s Set-A and Set-B, with average accuracy of 95.91% and 96.04%, respectively. The LieNet achieved 97% and 98% accuracy on the RL trail and MU3D datasets, respectively^27^.

In data-limited circumstances, Suzuki et al. devised a knowledge-transferred fine-tuning method for producing anti-aliased convolutional neural networks (CNNs). While fine-tuning the anti-aliased CNN, the authors applied knowledge from a pre-trained CNN that had not been overfitted to the restricted training data. To accomplish this goal, they use two forms of loss to transmit information: pixel-level loss for detailed knowledge and global-level loss for general detection knowledge. The ImageNet 2012 dataset findings reveal that the knowledge transferred to tuning yields high precision with hyper-parameter modifications^28^.

Zhang, presented an anti-aliased CNN model, which incorporates blur filters to normal the down sampling processes like stride convolution and pooling layers. The (lowpass) blur filter in the anti-aliased CNN eliminates such aliasing effects produced through down-sampling. As a result, anti-aliased CNNs outperform standard CNNs without blur filters in recognizing facial images. Based on this, numerous studies have refined the anti-aliased CNN and proven that blur filters work well for a wide range of visual recognition tasks, but also using it depends on the nature of the given task and the used data^5^. The following is a synopsis of past relevant work in Table 1.

**Table 1:** Summery of the related work.

Authors	Year	Publisher	Pros	Limitations & Challenges
Umer et al.^3^	2022	Springer	The trade-off between augmentation and deep features has an impact on the detection ability of the FER systems in unfamiliar test samples.	-
Zang^5^	2019	ICML conf.	Avoids aliasing issues caused by down sampling, demonstrating that blur filters perform effectively for an extensive variety of visual identification applications.	Efficiency depends on type of CNN architectures and the recognition task.
Chowdary et al.^9^	2021	Springer	Achieved higher performance after eliminating the fully connected layers from pre trained models.	The proposed model was using only one dataset in testing phase.
Abate et al.^10^	2022	Springer	Investigated the influence of masked faces on recognizing emotions pointing attention to the challenging occlusion problem.	Low FER accuracy which still needs to be improved.
Shaik et al.^11^	2022	Springer	A deep neural network was constructed that accepts both local and global attention information that outperformed many recent advances in FER.	Only operated on frontal pictures and was confined to real-time invariant face data.
Saurav et al.^12^	2022	Springer	Outperformed current CNN models in terms of computing efficiency and recognition accuracy.	Performed poorly in fear class.
Rajan et al.^13^	2020	IET Image Processing	Performed well in distinguishing surprised and joyful reactions.	Mis-classification in sadness and anger.
Khattak et al.^14^	2022	Springer	An effective method to extract age, gender, and emotions information from facial images.	Just one dataset was carried-out on gender and age experiments.
Bentomi et al.^15^	2022	Springer	avoid overfitting by using the early stopping criterion and also improved the overall accuracy.	The small size of the datasets
Liu et al.^16^	2021	IEEE	Utilization of face alignment to minimize the influence of ambient noise. Efficiently distinguishes between comparable sentiments such as fear and disgust.	The accuracy still needs to be improved.
Wang et al.^17^	2021	Wiley Online Library	Solve the problem of two tasks divergence (classification and regression).	Classification task and emotion recognition accuracy still need to be improved.
Taskiran et al.^18^	2020	Wiley	Useful for performing face recognition in videos extracted from systems that may contain images with illumination variations, noise, and blur while performing face recognition	The accuracy still needs to be improved for better face recognition.
Saurav et al.^19^	2021	Springer	The suggested model functioned well in identifying facial images in the neutral, surprise, disgust, and happiness classes	Struggled in the sad and afraid classes
Devi et al.^20^	2021	Springer	achieved significant classification accuracy	The major issue was the high training time
Li et al.^21^	2021	ScienceDirect	Overcome the overfitting problem that may occur during training phase	More images were needed to be collected to propose a better-optimized algorithm and also to make more improvements in facial recognition
Arora et al.^22^	2021	Springer	Achieved high classification accuracy.	Only one dataset in the testing phase was used
Zheng et al.^23^	2022	IEEE	Increase the capacity of instructors to recognize expressions in real-world world environments. A new dataset of intensity-based facial expressions known as EIDB-13 was generated.	For better feature extraction, the proposed approach has to be optimized further.
Fontaine et al.^22^	2022	Wiley Online Library	The model was beneficial in recognizing severe pain, and accurately predicted pain intensity.	The scientists did not compare the results to human observers’ assessments.
Lu et al.^23^	2022	ScienceDirect	Well recognized and identified the face and emotion in real time.	Limited to three emotions only (happy, regular, and unhappy).
Mohan et al.^6^	2020	IEEE	Succeeded to extract local characteristics from face images.	The high training time of this work hindered its performance
Mohan et al.^26^	2021	Springer	Simple architecture that can recognize and identify the face and emotion in real time.	Average recognition rates on all applied datasets.
Mohan et al.^27^	2021	IEEE	DCN model which used to accurately identify the multiscale variations of deceit.	-
Suzuki et al.^28^	2022	IEEE	Applying the anti-aliased CNN in data-limited situations achieving more accurate results.	Need to test model on more datasets for generalization.

## FER based deep learning architecture

Deep learning models have lately demonstrated more promising performance in FER than other conventional technologies, thanks to the availability of high-performance computing facilities, deep learning refers to " any training methodology capable of training a system with more than two or three non - linear hidden nodes.” The fusion of carefully weighted multiple layered data extraction makes it a better FER method in comparison to other strategies such as the Bayesian network, artificial-neural network (ANN), hidden-Markov model, support-vector machine, etc. The deep convolutional neural network (DCNN) is the most effective deep learning network for extracting features from facial expressions^1^.

### Convolutional neural network

CNN, also known as Conv, is a subtype of neural network that has become the most popular approach for computer vision due to its superior performance in handling images, videos, audio signals, and other visual inputs. A simple CNN is composed of three major layers: an input layer, one or more convolutions, and pooling levels, and a fully-connected-layer (FC). The data, in this case study, facial images, is passed to the CNN over the input layer and then travel through numerous hidden levels before reaching the output (FC) layer. The output layer demonstrates the network’s prediction in which facial expression is classified based on the output of the FER classifier. This output is compared to the real labels to evaluate the network performance. Deep CNN (DCN) has been commonly used and provided a more scalable approach in FER by adopting linear algebra methods, especially matrix multiplication, to find patterns in an image. While the CNN algorithm has made considerable progress in identifying facial expressions, numerous flaws remain, such as overly long training intervals that can be computationally intensive, the aliasing problem, and necessitate the use of graphics processing units (GPUs) to train models and low recognition rates in complicated scenarios^24^.

### Traditional CNN architectures

There are several classic CNN architectures, however, The FER model will concentrate and compare the proposed framework to (VGG_16_- VGG_19_- ResNet_50_- DenseNet_121_- InceptionV_3_- Xception -EfficientNetB_0_). In the next section, a brief discussion of the CNN models will be mentioned from the viewpoint of this study.

- ***VGG***: VGG comes in two versions (16 and 19). VGG-19 optimizes the network by replacing larger kernel-size filters with several (3 × 3) kernel-size filters, one after the other.
- ***ResNet***_***50***_: ResNet_50_ improved the CNN conceptual design by including the concept of residual understanding, often known as an “identity shortcut connection’’, which allows the network to be trained on hundreds of layers without compromising performance.

1 $$\:{F}_{m+1}^{K}={y}_{\mathfrak{c}}\left({F}_{1\to\:m}^{k}\:,{K}_{1\to\:m}\right)+{F}_{1}^{k}\ge\:1\:\:\:\:\:\:\:\:\:\:\:\:\:\:$$

2 $$\:{F}_{m+1}^{k}={y}_{\mathfrak{a}}\left({F}_{\mathcalligra{m}+1}^{k}\right)\:\:\:\:\:\:\:\:\:\:\:\:\:\:\:\:\:\:\:\:\:\:\:\:\:\:\:\:\:\:\:\:\:$$

3 $$\:{y}_{\mathfrak{c}}\left({F}_{1 \to m}^{k},\:{K}_{1 \to m}\right)={F}_{\mathcalligra{m}+1}^{k}-{F}_{1}^{k}\:\:\:\:\:\:\:\:\:\:\:\:\:\:\:\:\:\:\:\:\:\:\:\:\:\:\:$$

where $\:{\:F}_{1}^{k}$is an input of the l^st^ layer and $\:{y}_{\mathfrak{c}}\left({F}_{1\to \mathfrak{m}}^{k}\right)$ is the transformed signal which produces a cumulative output $\:{F}_{\mathfrak{m}+1}^{k}$ that is then provided to the next layer after integrating with the activation function $\:{y}_{\mathfrak{a}}$.

- ***DenseNet***: it is a convolutional network with every layer attached to all other deep layers in the network, Dense-Net was designed primarily to reduce the vanishing-gradient loss and decline in accuracy in high-level neural networks.
- ***Inception-V***_***3***_: The goal of Inception(V_3_) aimed to decrease the computational complexity of deep-networks while maintaining generalization.
- ***Xception***: Xception is a deep CNN architecture with depth-wise separable convolutions.
- ***EfficientNet***: It is a scaling method that employs a compounded coefficient to equally scale all depth, width/resolution dimensions.

## Algorithm 1: proposed DCN overview

**Fig. 2:**
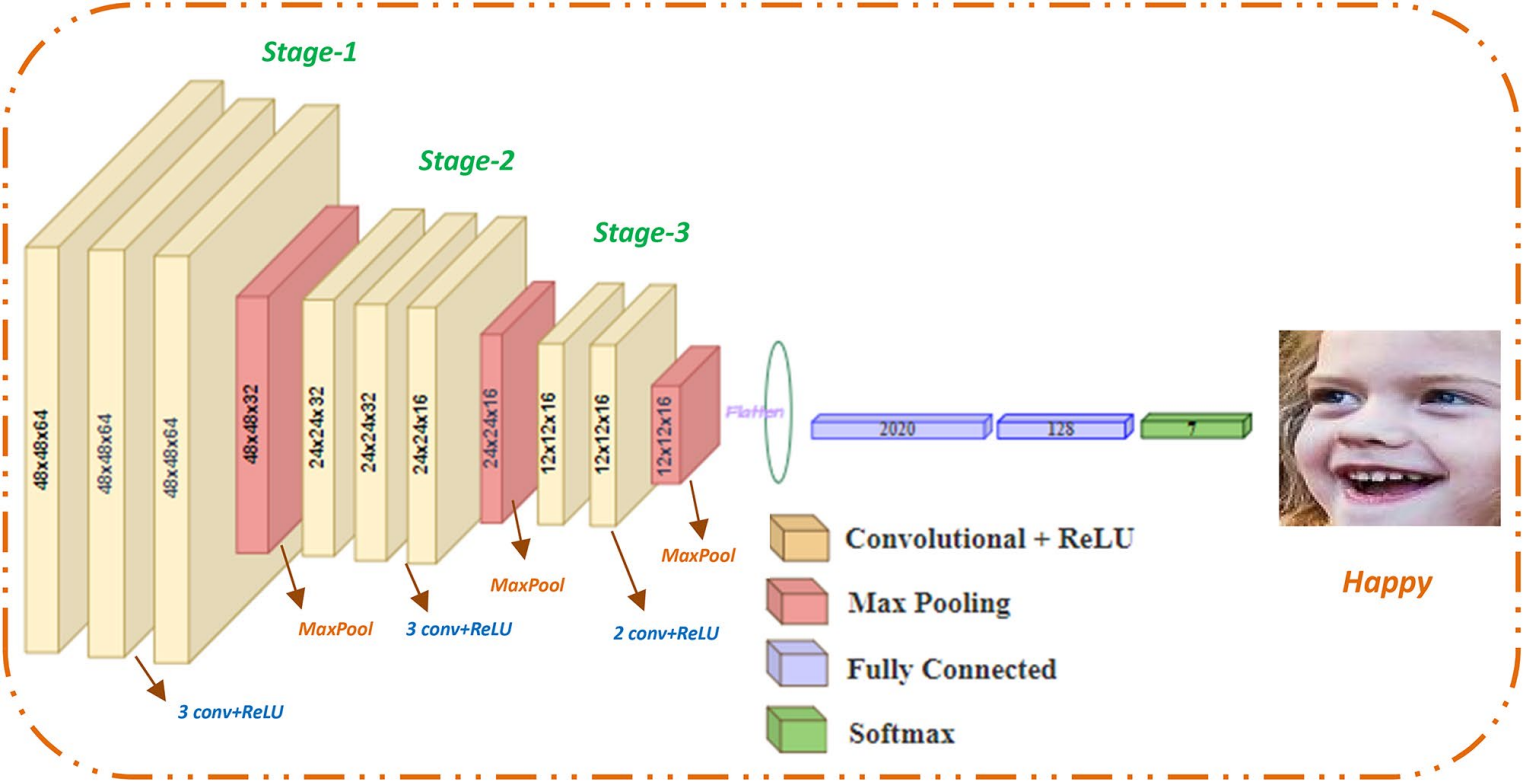
Detailed kernel structure of the proposed DCN model.

This study demonstrated two effective FER systems that can identify up to eight different facial expressions. (Angry, neutral, surprise, happy, sad, disgust, contempt, and fear). The input to the system is an image containing a facial region with a specific expression as input. The first provided DCN approach is made up of several convolutional, dropout, and dense layers. Using a combination of mixing, matching, and layering to develop an optimal structure that outperforms previous architectures. The convolutional model’s whole layout is revealed in Fig. 2.

Following the first layer, three convolutional layers (Stage-1). For each input channel, the convolution technique generates numerous feature maps. Subsequently, another three convolutional layers (Stage-2) have been performed; the fourth and fifth convolutional layers have 32 filters, and the six conv. has 16 filters. Another two convolution layers (Stage-3) are conducted, both having 16 filters. Kernels of size 3$\:\times\:$3, and one stride are employed in all the convolutional layers with no padding. The convolution operation can be expressed using Eq. 4^29^.

4 $$\:{\:\:F}_{m}\left(m,n\right)={\text{C}\text{o}\text{n}\text{v}2\text{D}\left(\text{{\rm\:I}},K\right)}_{mn}=\sum\:_{t=1}^{s}\sum\:_{z=1}^{s}{K}_{\left(t,z\right)\:\times\:\:}{I}_{\left(t+m,\:\:z+n\right)\:}+b\:\:\:\:\:\:\:\:\:\:\:\:\:\:\:\:\:\:\:\:\:\:\:\:\:\:\:\:\:\:\:\:\:\:\:\:\:\:\:\:\:\:\:$$

where $\:{F}_{m}\left(m,n\right)$ is the convolution value in the resulting feature map at location$\:\left(\mathfrak{m},\mathfrak{n}\right)$, I is an input image, b is the bias, and k is the kernel with rows (t), columns (z), and size (s).

A batch normalization layer was added to speed up the training process, and a max pooling layer is placed after each convolutional layer Stage (1, 2, and 3). Each of the three pooling layers has kernel sizes of 2 × 2 and with stride step = 2. The maximum pooling layers are employed for down sampling. Equations (5), (6) define the output of the feature map size following the pooling process^29^.

5 $$\:\:\:\:\:\:\:\:\:\:\:\:\:\:\:\:\:\:\:\:\:\:\:\:\:\:\:\:\:\:\:\:\:\:\:\:\:\:\:\:\:\:\:\:\:\:\:\:\:\:\:\:\:\:\:\:\:\:\:\:\:\:\:\:\:\:\:\:\:\:\:\:\:\:\:\:\:\:\:\:\:\:W^{\prime}=\:\frac{W-p}{s}\:\:\:\:$$

6 $$\:\:\:\:\:\:\:\:\:\:\:\:\:\:\:\:\:\:\:\:\:\:\:\:\:\:\:\:\:\:\:\:\:\:\:\:\:\:\:\:\:\:\:\:\:\:\:\:\:\:\:\:\:\:\:\:\:\:\:\:\:\:\:\:\:\:\:\:\:\:\:\:\:\:\:\:\:\:\:\:\:\:h^{\prime} = \:\frac{h-p}{s}\:\:\:\:\:\:$$

where$\:\:W^{\prime}$ and $\:h^{\prime}$ are the output feature map’s height and width, $\:W$ and $\:h$ are the input feature map’s height and width, $\:p$ is the pool dimensions, and $\:s$ is the pooling layer’s stride size. The formula in Eq. (7) was used to calculate the feature map’s size after each convolution operation^29^.

7 $$\:{\:\:\:\:\:\:\:\:\:\:\:\:\:\:\:\:\:\:\:\:\:\:\:\:\:\:\:\:\:\:\:\:\:\:\:\:\:\:\:\:\:\:\:\:\:\:\:\:\:\:\:\:\:\:\:\:\:\:\:\:\:\:\:\:\:\:\:\:\:\:\:F}_{Output}=\frac{L-K+2P\:}{s}\:\:\:\:\:\:\:\:\:\:\:\:\:\:\:\:\:\:\:\:\:\:\:\:\:\:\:\:\:\:\:\:\:\:\:\:\:\:\:\:\:\:\:\:\:\:$$

where $\:L$ stands for the given input size,$\:\:K$ for the number of kernels within each layer,$\:\:P$ for padding, and $\:s$ for stride size.

Afterward, a fully connected network is applied that is composed of two dense layers (2020 and 128), respectively. Dropout is applied to regulate the convolutional layers, with 0.7 chance of preserving every neuron. Throughout the network, a ReLU activation function is employed, the Adadelta, SGD, and Adam optimizers have all been used in tunning phase and for optimizing the hyperparameters; however, Adam delivers the best results, hence it has been utilized. The loss is being evaluated with the categorical cross-entropy-function. The output layer is composed of seven units to detect the seven facial expressions. In the final dense layer, the SoftMax function has been applied as an activation function to produce the most frequent class of input data through the classification phase. The mathematical equation of the ReLU function can be computed by Eq. 8^29^.

8 $$\mathcal{F}\left(\text{x}\right)=\mathfrak{m}\mathfrak{a}\mathcalligra{x}\left(0,\text{x}\right)\:\:\:\:\:\:\:\:\:\:\:\:\:\:\:\:\:\:\:\:\:\:\:\:\:\:\:\:\:\:\:\:\:\:\:\:\:\:\:\:\:\:\:\:\:\:\:\:\:\:\:\:\:\:\:\:\:\:\:\:\:\:\:\:\:\:\:\:\:\:\:\:\:\:\:\:\:\:\:\:\:\:\:\:\:\:\:\:\:$$

The goal from this DCN model is not only to determine the best effective network for the model but rather to compare the classification capabilities of various traditional CNN models on different well-known datasets. This is why the core idea behind the architectural selections relies on a somewhat regular network design premised on well-known regularization methods, tiny kernels and a smooth set of hyper-parameters. Also providing a dropout layer will settle the learning. Figure 2 illustrates the layering structure of each stage. The output features from each stage will be the input to the following stage, i.e., the output of stage-1 will be the input to stage-2, and so on. The next Algorithm illustrates the applied procedures on the proposed DCN methodology.

**Algorithm 1:**
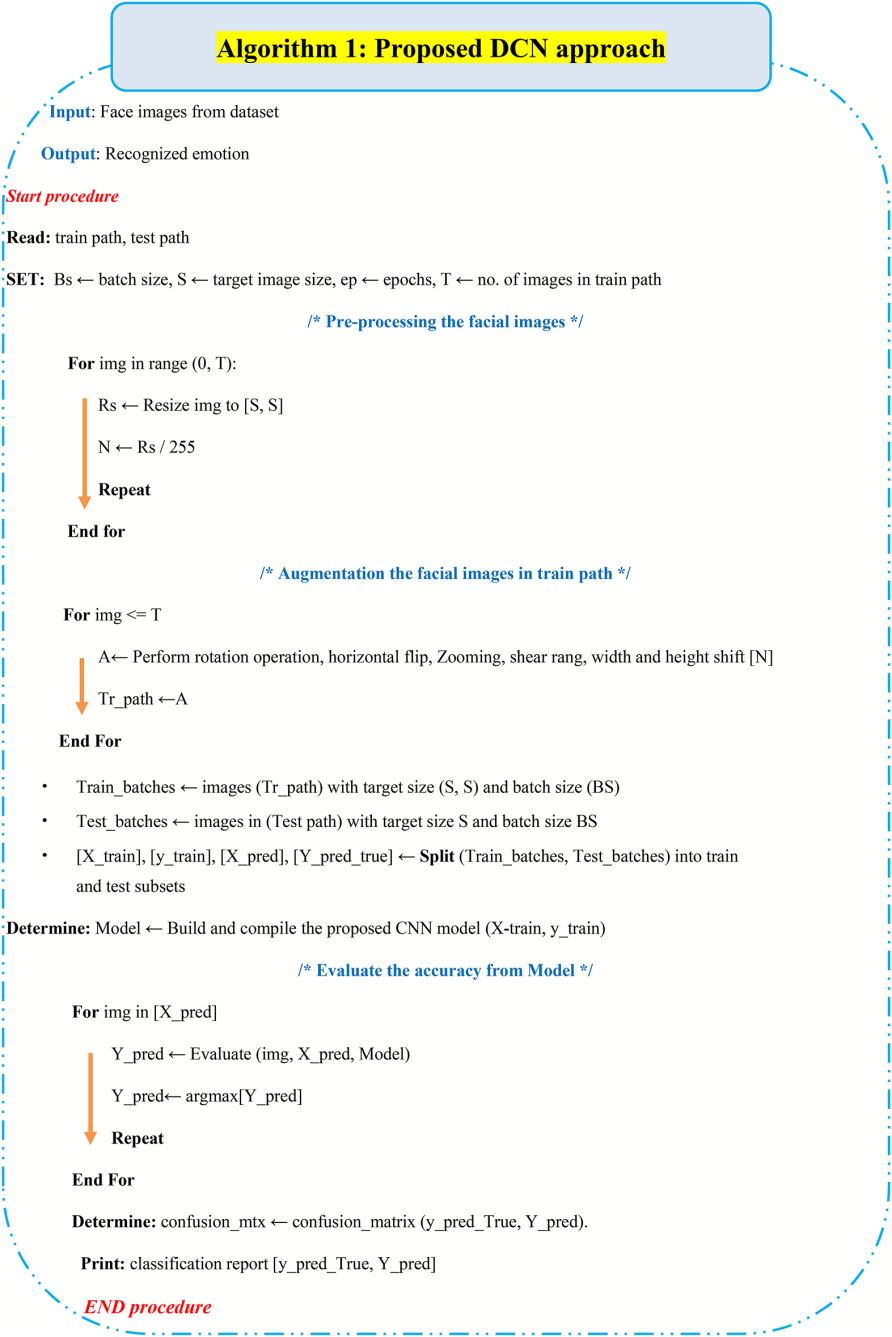
Detailed kernel structure of the proposed DCN model.

## **Experiment procedure**

**Fig. 3:**
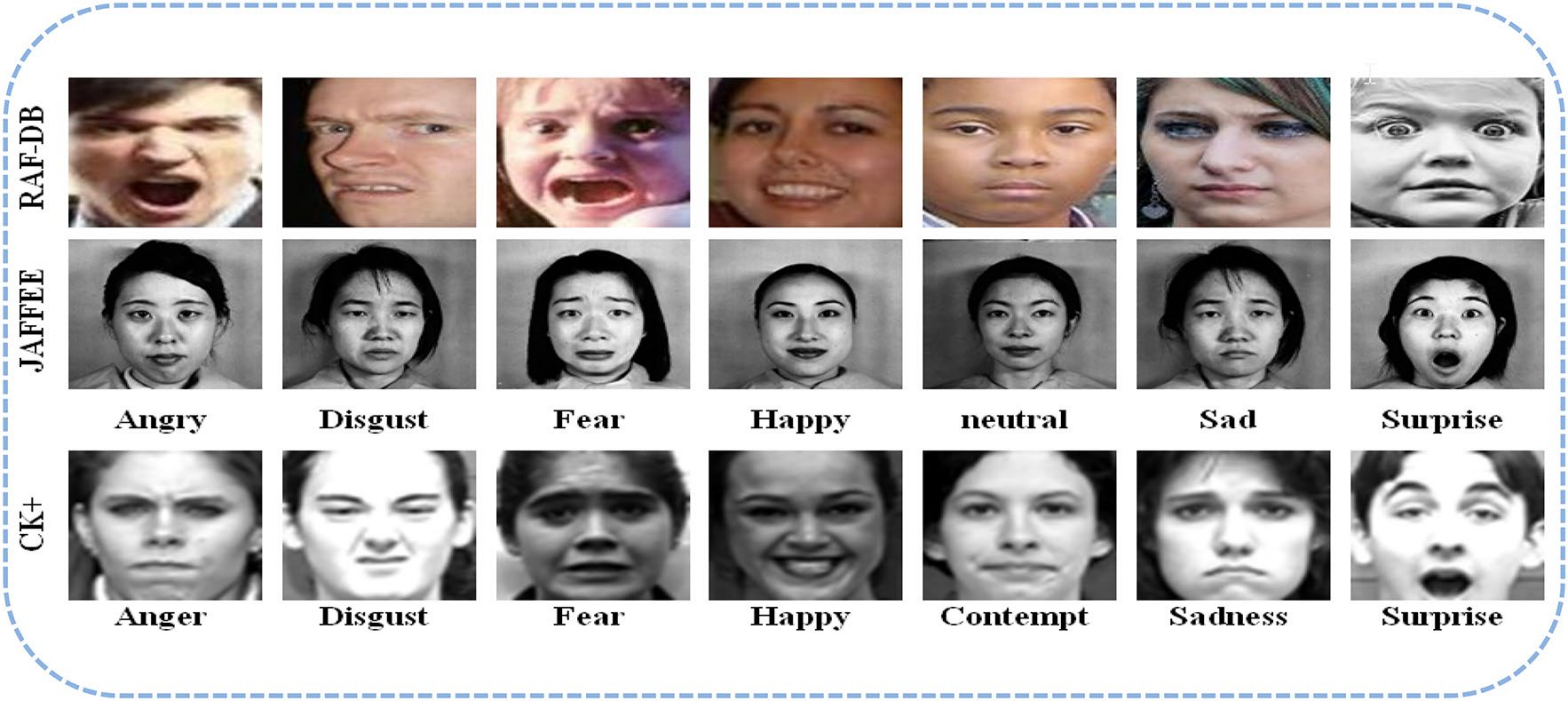
Samples from RAF-DB, JAFFEE, and CK plus datasets.

The emotion data has been employed from a standardized dataset to train the facial emotion recognizer in the proposed framework and then evaluated using data from the remaining dataset that wasn’t included in the training set. As a consequence, the following three key phases of interest have been accomplished: preprocessing and augmentation of the facial-data, implementation of all deep learning techniques. The Detailed structure of the suggested deep CNN model is demonstrated in Fig. 3. The primary goal is to estimate the performance of all facial expressions (disgust, angry, fear, happy, sad, neutral, contempt and surprise) in the datasets as mentioned before.

### Data sets description

This study will make use of three well-known, freely accessible databases of real-world facial emotions: RAF-DB, JAFFEE, and CK-Plus. The datasets are provided in References^31–33^. These datasets provide a variety of complex scenarios as well as unbalanced samples for various condition states. The details are as follows:

**Table 2:** Strength and gap between RAF-DB, JAFFE and CK + datasets.

Data set	Strength	Gap
**RAF_DB**	A real world dataset with real world facial images occlusion problems (masks, cigarettes, hair on face, etc.) Different illuminations, pose, gender, etc.) Available on line.	High variety in (occlusions, illuminations, gender, ages, …) may affect the training phases. Very Low in images quality.
**JAFFE**	High-resolution images. High level of standardization (lighting and facial positioning). Available on line.	Relatively small. Produced in a laboratory setting.
**CK+**	High-quality images. The images were all taken under controlled circumstances (lighting, pose, and facial expression) are consistent across all images. Detailed annotations. Available on line.	Limited size of data subjects Standardized laboratory settings were used for all pictures. May not accurately reflect how people express their feelings in everyday situations.

**Fig. 4:**
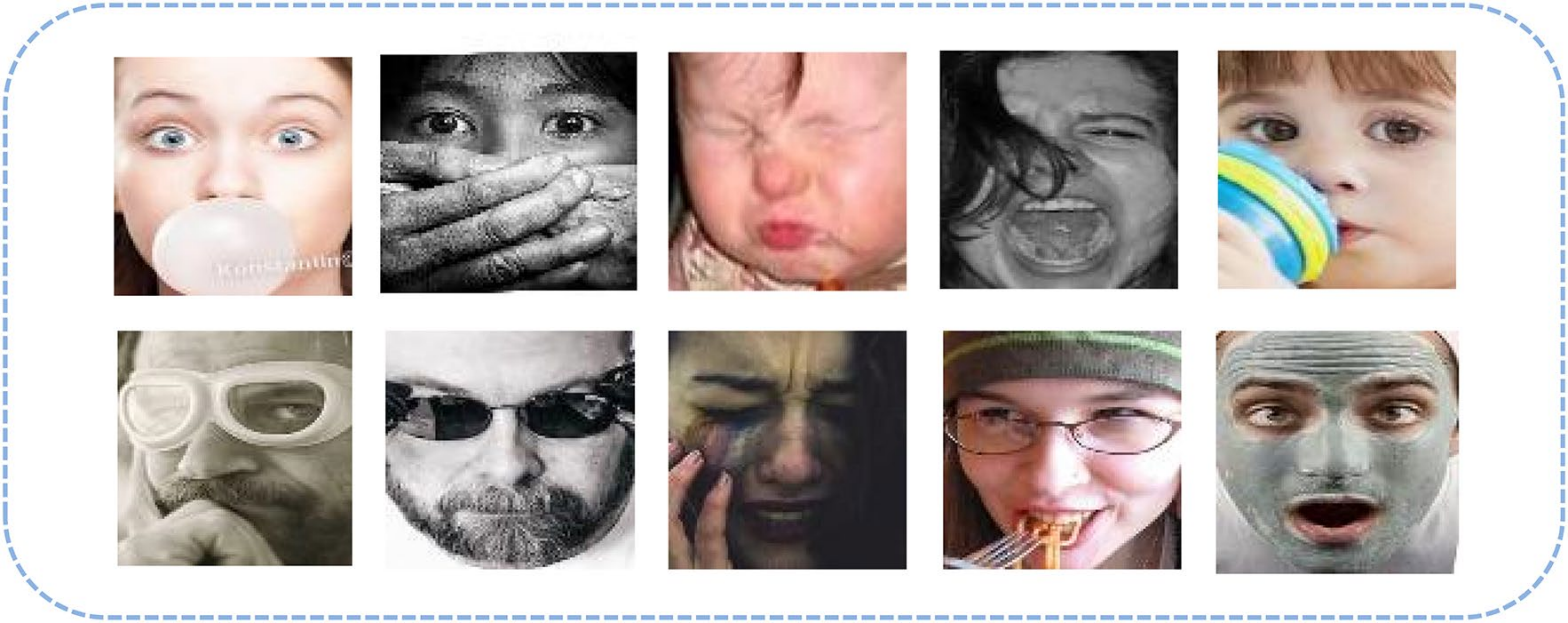
Samples of challenges in RAF dataset.

#### RAF_DB (Real-world affective face database)

It is a real-world database with different real-world problems, such as those involving masks, smoking, hair covering the eyes, etc. Some of the challenges in this dataset is shown in Fig. 4. In addition to the deferent poses and illumination, thus it can be considered as one of the most challenging facial datasets to deal with.

The RAF dataset was gathered and built using the Internet and labelled using crowdsourcing. It is divided into two types of expression groups: seven basic and eleven compounds. This experiment places an emphasis on basic emotion recognition issues and is tagged with six basic emotion subjects (surprise, happy, sad, disgust, fear, and angry) along with (neutral) expression. Especially, the RAFDB contains 15,339 basic emotion images, all 100 × 100 RGB images, of which approximately 80% (i.e., 12,271 images) are used in training phase and 20% (i.e., 3068 images) are used in testing phase. A significant number of them are aligned facial images with relatively low resolution.

#### JAFFE (Japanese female facial expressions)

This is a commonly used dataset for facial expressions, typically consisting of 213 grayscale images with a resolution of 256 × 256 from 10 Japanese women. Each individual continues to pose for three to four basic expressions as well as the neutral mood. This dataset is challenging to analyze because it provides only few samples of facial expression images.

#### CK+ (Cohn-Kanade extending)

CKplus, is a more detailed version of the original CK dataset (Kanade et al., 2000, frequently used for expressions recognition tasks and contains 593 frame sequences ranging in age from 18 to 50 years old, with a variety of genders and culture. Out of these samples, 327 sequences involving 118 various individuals have been annotated. These emotions include anger, sadness, happiness, contempt, disgust, surprise, fear and neutral, with a resolution of 48 × 48 and in PNG format. All of these image sequences were captured in a laboratory-controlled setting. CK + dataset has been augmented to increase its size to became 4,021images. Figure 3 displays a sample of facial images involving multiple facial emotions from the three Datasets also illustration of their strength and gap are summarized in Table 2 and a summarization of them is shown in Table 3.

### Dataset preprocessing

Data preparation is a vital step in computer vision. The term “preprocessing " refers to all of the adjustments that occur on actual data prior to delivery to the deep CNN-model. First initial stage is to upload all the required libraries in the preprocessing phase, such as NumPy, Matplotlib, and Pandas.

Jaffe dataset has been rescaled to 128 × 128 in JPG format. Following that, various augmentation procedures such as (image rotation, zooming, width& height shifting, shear mapping, horizontal and vertical flipping, and bright enhancement) on (CK+, JAFFEE, and RAF) have been carried out to boost the capacity of input datasets and balance them to deliver more accurate and faster performance using the suggested DCN approach. Aside from that, Ref^33^ provides details on these augmentation techniques. Table 3 show the new distribution of facial datasets after augmentation.

**Table 3:** Facial data distribution over several distinct emotions in augmented RAF-DB, JAFFE and CK+.

Dataset	Total no. of facial images	Dataset distribution		Image Size	Format
		Train	Validation		
**RAF_DB**	12,845	75%	25%	JPG	(100 × 100) RGB-images
**JAFFE**	4489	75%	25%	JPG	(128 × 128) grayscale-images
**CK+**	4021	90%	10%	PNG	(48 × 48) grayscale-images

**Fig. 5:**
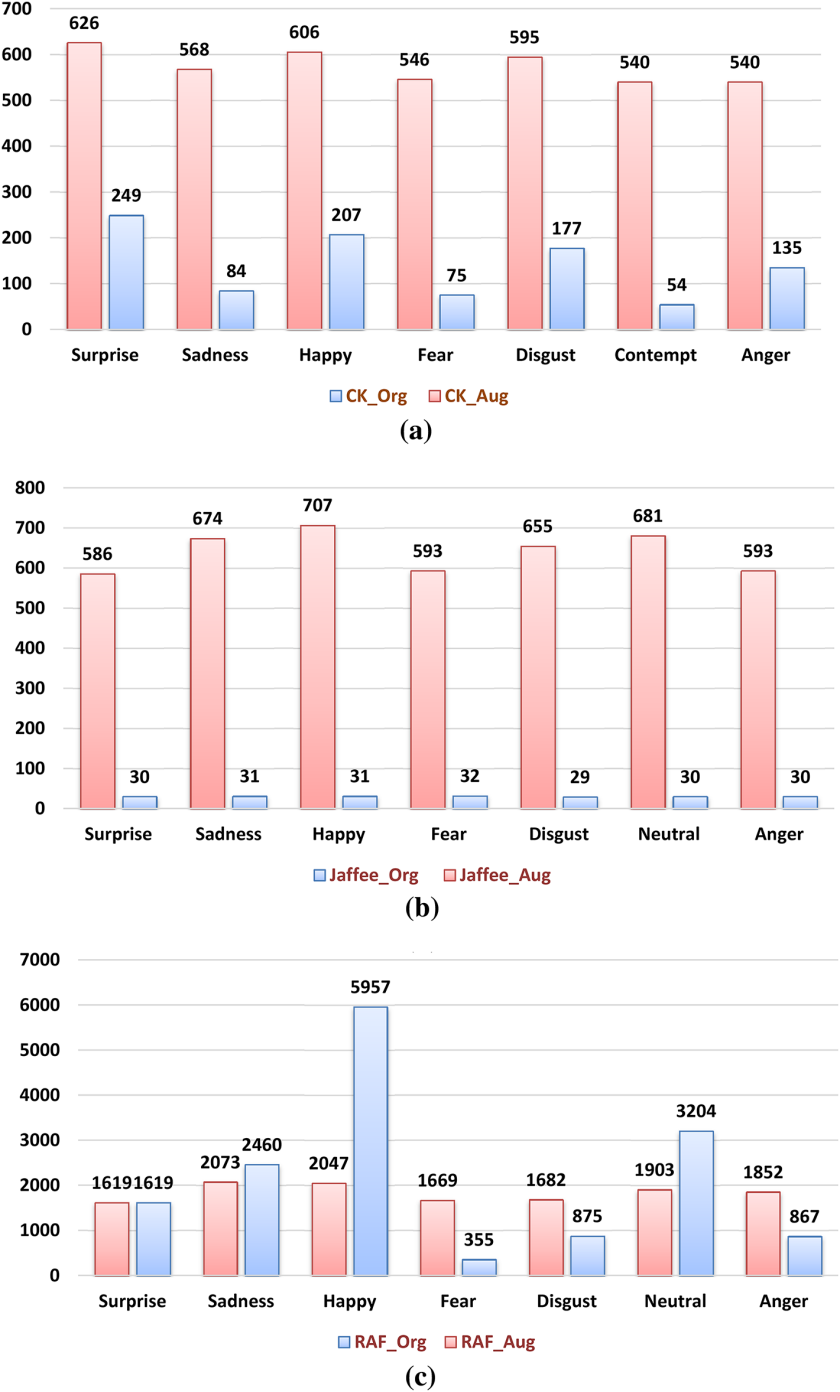
(a) CK+, (b) Jaffee datasets before and after augmentation, and (c) RAF-DB class distribution.

By increasing the dataset size, augmentation can assist in making the model perform better by decreasing overfitting. When a model learns the patterns in the training data too effectively yet lacks the ability to generalize to new data, overfitting occurs. By generating new data that is similar to the training data but not identical, augmentation can aid in the reduction of overfitting. As opposed to learning the precise patterns in the training data, this can assist the model in learning the general patterns in the data. Also, balancing the datasets will improve the accuracy of identifying each emotion correctly, so it is an important preprocessing step. Figure 5 illustrates the effect of augmentation on Ck+, JAFFE, and RAF-DB. The final step is dividing the dataset into train and test sets to be delivered to the deep CNN-model.

Keras ImageDataGenerator has been employed for augmenting the three datasets since it offers many significant advantages: adopting a generator architecture for data augmentation offers customized, consistent, and efficient augmentation with little code as opposed to manual techniques, permitting enormous versatility to improve CNN training diversity.

### Evaluation metrics

The effective performance of facial emotion recognition is regarded by evaluating the necessary precision, computing time, and complexity level. It can be counted how parameter changes might impact the model’s performance during the training process by delving into deep learning techniques. The most widely used performance measurements are confusion matrix, recall, and accuracy^34^.

The four values that must be provided by the assessment techniques are true positives ($\:{T}_{\mathcal{P}}$), false positives ($\:{F}_{\mathcal{P}}$), true negatives ($\:{T}_{\mathcal{N}}$), and false negatives ($\:{F}_{\mathcal{N}}$). The number of classes drops to two: $\:{T}_{\mathcal{P}}$ and $\:{T}_{\mathcal{N}}$ when an activity is appropriately defined. It can be $\:{F}_{\mathcal{P}}$ or $\:{F}_{\mathcal{N}}$ when an activity is incorrectly categorized.

- ***Precision***: demonstrates the model’s performance on the testing set. It depicts the number of models that properly anticipated from all positive categories. It can be calculated from Eq. (9)^35^.

9 $$\:\:\:\:\:\:\:\:\:\:Precision=\frac{{T}_{\mathcal{P}}}{{T}_{\mathcal{P}}+{F}_{\mathcal{P}}}\:\:\:\:\:\:\:\:\:\:\:\:\:\:\:\:\:\:\:\:\:\:\:\:\:\:\:\:\:\:\:\:\:\:\:\:\:\:\:\:\:\:\:\:\:\:\:\:\:\:\:\:$$

- ***Sensitivity*** (Recall): this metric represents the number of positive samples that have been appropriately labeled as true positives and can be measured by Eq. (10)^36^.

10 $$\:Recall=\:\frac{{T}_{\mathcal{P}}}{{T}_{\mathcal{P}}+{F}_{\mathcal{N}}}\:\:\:\:\:\:\:\:\:\:\:\:\:\:\:\:\:\:\:\:\:\:\:\:\:\:\:$$

- ***F1-score***: it is a metric that incorporates sensitivity together with precision, and it is calculated from Eq. (11)^37^.

11 $$\:F-score=2\:\times\:\:\frac{Precision\:\times\:Recall}{Precision\:+Recall}\:\:\:\:\:\:\:\:\:\:\:\:\:\:\:\:\:\:\:\:\:\:\:\:\:\:\:\:\:$$

- ***Accuracy***: What is meant by accuracy is the number of correctly detected instances. Accuracy is determined by dividing the overall number of right classes by the sum of the classes, It is computed from Eq. (12)^37^.

12 $$\:Accuracy=\:\frac{{T}_{\mathcal{P}}+{T}_{\mathcal{N}}}{{T}_{\mathcal{P}}+{T}_{\mathcal{N}}+{F}_{\mathcal{P}}+{F}_{\mathcal{N}}}\:\:\:\:\:\:\:\:\:\:\:\:\:\:\:\:\:$$

## Results and discussion

In this research, several experimental studies using various deep-learning models have been included. First study analyzed classical CNN networks such as Inception, VGG, ResNet, and other previously discussed architectures, while the second experiment concentrated on the proposed DCN model (Algorithm-1).

Following that the experimental studies of the proposed AA-DCN model (Algorithm-2). The last one compares the proposed FER model against typical topologies. These experiments have been applied on the three datasets specified in Sect. 5.1. To evaluate the proposed DCN model, the following metrics have been utilized: confusion matrix, recall, precision, and F1 score.

All tests were carried out on a laptop with the requirements given: Microsoft Windows-10 operating system, Intel(R) Core (TM) i7-7600U CPU @ 2.80 GHz 2.90 GHz, 8 GB of RAM, Intel HD Graphics 620. In addition, the experimental results have been evaluated on Kaggle API, with GPU100.

These parameters have been assigned after a lot of trials to in the training phase to reach to the best recognition rate with the minimal computing time. The parameters are assigned to the provided values shown in Table 4 with batches of 32. Relative to other sizes, this predefined value is appropriate for the learning process and doesn’t require a lot of computing time to work out. Furthermore, the number of epochs in the of training phase has been scaled to 30 which gave the best performance, after evaluating a variety of other values from 10 to 100. The learning rate has been fixed at 0.0006. The TensorFlow framework and a Google open-source deep learning library are used to implement the suggested network.

**Table 4:** The tuning parameters.

Parameters	Value
Epochs	30
Batch size	32
Learning rate	6 × 10^**− 3**^
Optimizer	Adam
Loss function	Spars_ categorical

### Evaluation of classical CNN on CK + dataset

In this work, some of the most popular and impactful types of pre-trained models that are often used for image classification tasks with their best hyperparameters have been adopted as starting points to compare them with the outcomes of the proposed methods. These classical models are deep learning models with complex neural networks that’ve already been trained on a massive and diverse dataset, learning general patterns and features, and are ready to be used or fine-tuned for a specific task. All these models have been evaluated on the three datasets using the hyper parameters in Table 7.

The capability of CNN has been shown by deploying seven alternative architectures; ResNet_50_^28^, Inceptionv_3_^38^, VGG^34^, EfficientNet^39^, Xception^40^, and DenseNet^41^. Training augmented CK + datasets have been utilized with those previous architectures.

**Fig. 6:**
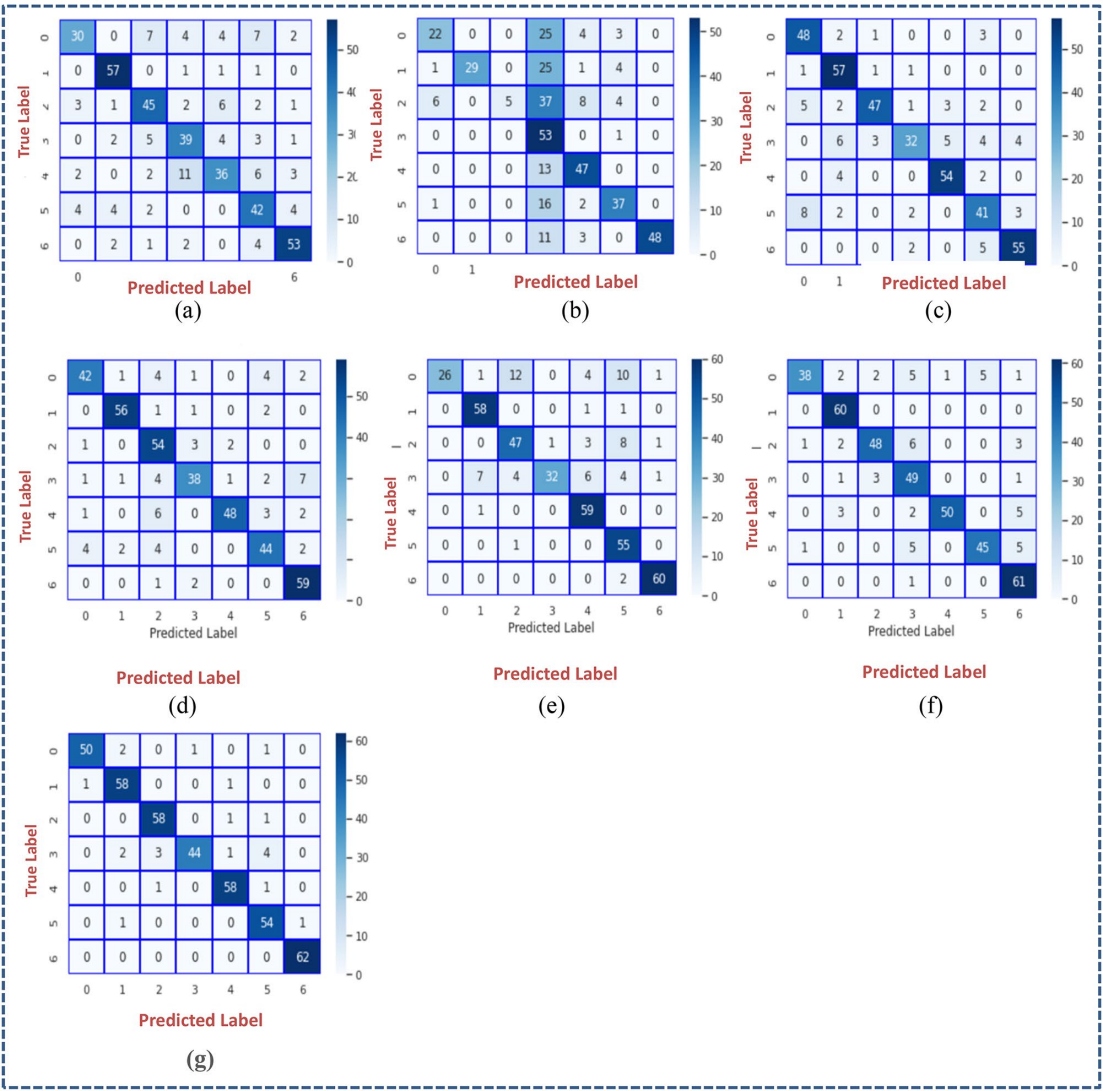
Confusion matrix of classical CNN models: (a) Densenet121, (b) InceptionV3, (c) VGG16, (d) VGG16, (d) VGG19, (e) ResNet50, (f) Xception, and (g) EfficientNetB0.

The performance of each CNN model is shown in Table 5. Table 6 illustrates the classical CNN model’s strengths and gaps. Confusion matrix of classical CNN models is depicted in Fig. 6. Hyperparameter Settings of traditional CNN Models is illustrated in Table 7. DenseNet_121_ had the lowest precision at 74% then InceptionV3: 77% and the findings began to improve in each architecture of VGG_16_: 83%, ResNet_50_: 87%, VGG_19_: 88%, Xception: 91%, with the best accuracy being about 93% basing on EfficientNetB_0_.

**Table 5:** Classical CNN architectures classification report on augmented CK + dataset.

DenseNet121			InceptionV3			VGG16			VGG19		
Precision	Recall	f1-score	Precision	Recall	f1-score	Precision	Recall	f1-score	Precision	Recall	f1-score
0.76	0.59	0.67	0.71	0.63	0.67	0.69	0.89	0.77	0.90	0.81	0.85
0.88	0.93	0.90	0.96	0.83	0.89	0.88	0.85	0.86	0.78	0.98	0.87
0.67	0.73	0.70	0.54	0.82	0.65	0.85	0.87	0.86	0.79	0.90	0.84
0.79	0.61	0.69	0.66	0.80	0.72	0.82	0.76	0.79	0.89	0.76	0.82
0.73	0.82	0.77	0.95	0.68	0.80	0.89	0.82	0.85	0.94	0.82	0.87
0.56	0.84	0.67	0.80	0.79	0.79	0.83	0.70	0.76	0.85	0.84	0.85
1.00	0.66	0.80	0.98	0.84	0.90	0.88	0.92	0.90	0.93	0.90	0.92
Overall	accuracy	0.74	Overall	accuracy	0.77	Overall	accuracy	0.83	Overall	accuracy	0.86

ResNet50			Xception			EfficientNetB0					
Precision	Recall	f1-score	Precision	Recall	f1-score	Precision	Recall	f1-score			
0.68	0.93	0.78	0.82	0.93	0.87	0.91	0.98	0.95			
0.96	0.92	0.94	0.95	0.95	0.95	0.97	0.98	0.98			
0.83	0.82	0.82	0.93	0.85	0.89	0.94	0.78	0.85			
0.85	0.85	0.85	0.91	0.89	0.90	0.82	0.93	0.87			
1.00	0.93	0.97	1.00	0.93	0.97	0.98	0.93	0.96			
1.00	0.64	0.78	0.93	0.89	0.91	0.92	0.96	0.94			
0.89	1.00	0.94	0.88	0.95	0.91	0.98	0.95	0.97			
Overall	accuracy	0.87	Overall	accuracy	0.91	Overall	accuracy	0.93			

**Table 6:** Strengths and gaps in traditional CNN models.

	Accuracy	Strength	Gap
DenseNet121	74%	Reduce overfitting, increase feature reuse, reduce the number of parameters	The massive increase in parameters caused by an increase in the number of feature maps in every layer
InceptionV3	77%	End up replacing large - sized filters with (1 × 7), and (1 × 5) filters	Complicated structure. Lack of consistency.
VGG16	83%	Max Pooling has been applied	The use relatively demanding fully - connected layers
VGG19	88%	More powerful than VGG16	More complex, more computationally intensive potentially more prone to overfitting.
ResNet50	87%	The concept of residual learning was introduced. Error rate for deeper networks has been lessened	Little-complex architecture
Xception	91%	More efficient in terms of processing time than standard convolutions.	Depth-wise separable convolution layer replaces the typical Inception modules
EfficientNetB0	93%	Employs a compounded coefficient to scale all depth/width/resolution elements similarly	Slow training at the large image resolution

**Table 7:** Hyperparameter settings of traditional CNN models.

Model	Optimizer.	Lr. Rate	Loss Func.	Batch
ResNet50	SGD	0.1 (with decay)	Cross-entropy	32
Inceptionv3	RMSprop	0.001	Cross-entropy	32
VGG16	SGD	0.01 (with momentum)	Cross-entropy	32
VGG19	SGD	0.01 (with momentum)	Cross-entropy	32
EfficientNetB0	Adam	0.001 (with warmup)	Cross-entropy	32
Xception	Adam	0.001	Cross-entropy	32
DenseNet121	SGD	0.1 (with warmup and decay)	Cross-entropy	32

### The evaluation of the provided DCN on CK + dataset

By observing the pros and cons of the seven CNN architectures stated previously, two robust models have been suggested that unifies all effective layers. The first DCN model (Algorithm-1) has been tested to analyze the effect of using augmentation and stride layers after effectively fine tuning the model’s hyperparameters as illustrated in Sect. 4.

**Fig. 7:**
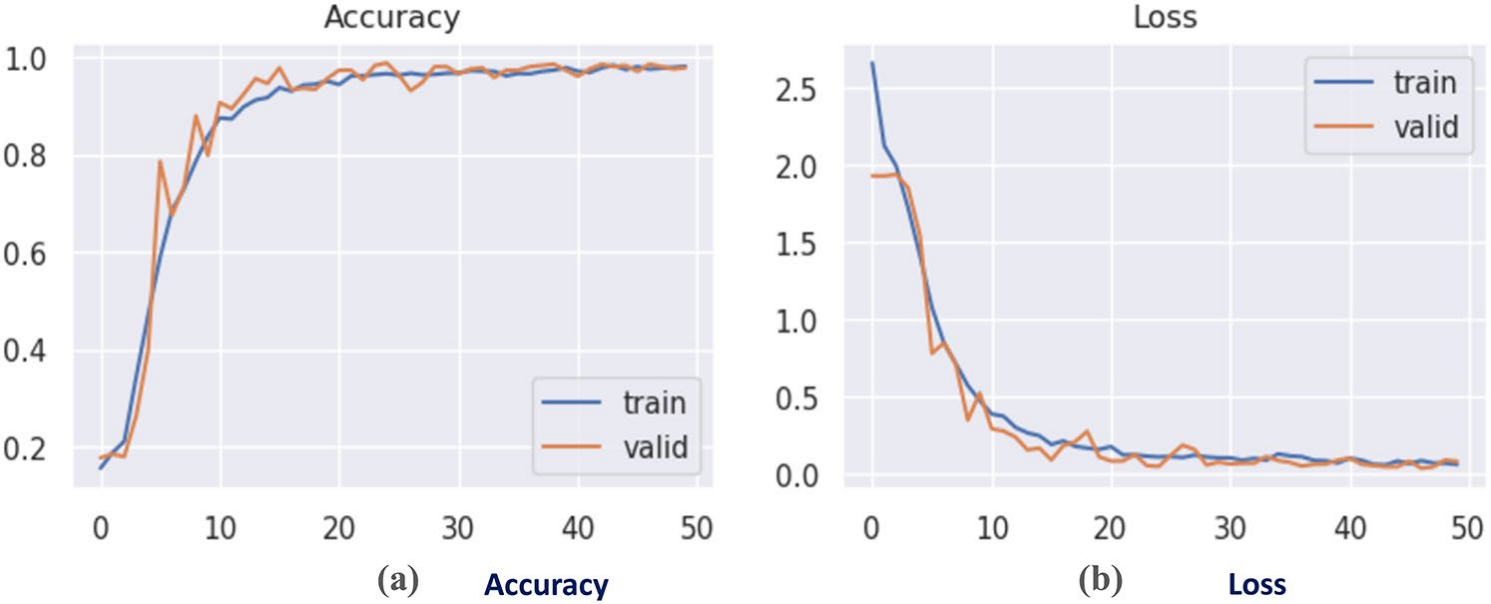
(a) Accuracy curve, (b) Loss curve for proposed CNN on CK + dataset.

**Fig. 8:**
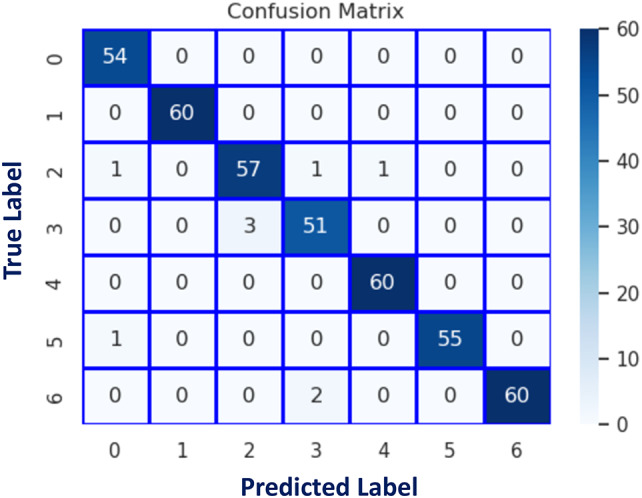
Confusion matrix using (CK+).

The DCN was initially evaluated on the augmented CK + dataset after dividing it into two sets: train set, and test set; with a 9: 1 ratio. The model accuracy and loss curves are seen in Fig. 7. The confusion matrix of the suggested FER model is illustrated in Fig. 8. Table 8shows the classification report.

It has been proven that the recommended DCN model employing the CK + dataset has performed the best, with a training accuracy of 98.09%, while the validation accuracy has reached about 98.32% in only 3.32 min, achieving the best computational time with the highest recognition rate compared to the classical deep CNNs.

**Table 8:** Proposed DCN classification-report (CK+).

Label	Emotion	Proposed DCN		
		Precision	Recall	F1-score
0	Anger	0.96	1.00	0.98
1	Contempt	1.00	1.00	1.00
2	Disgust	0.95	0.95	0.95
3	Fear	0.94	0.94	0.96
4	Happy	0.98	1.00	0.99
5	Sadness	1.00	0.98	0.99
6	Surprise	1.00	0.97	0.98
**Over all accuracy**				0.98

### Evaluation of proposed DCN model utilizing more datasets

This experiment aims to test and evaluate the FER model’s performance by using (2) different-datasets in order to highlight the potential of the provided CNN method.

#### Using the Jaffee dataset

**Fig. 9:**
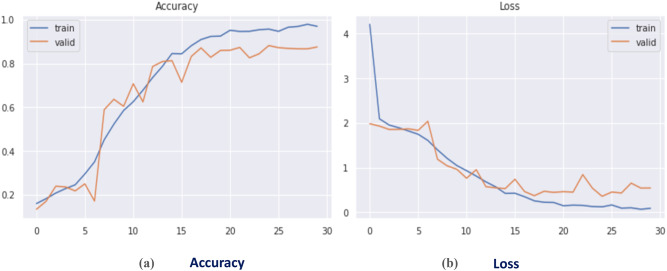
(a) Accuracy curve (b) Loss curve using Jaffee dataset.

**Fig. 10:**
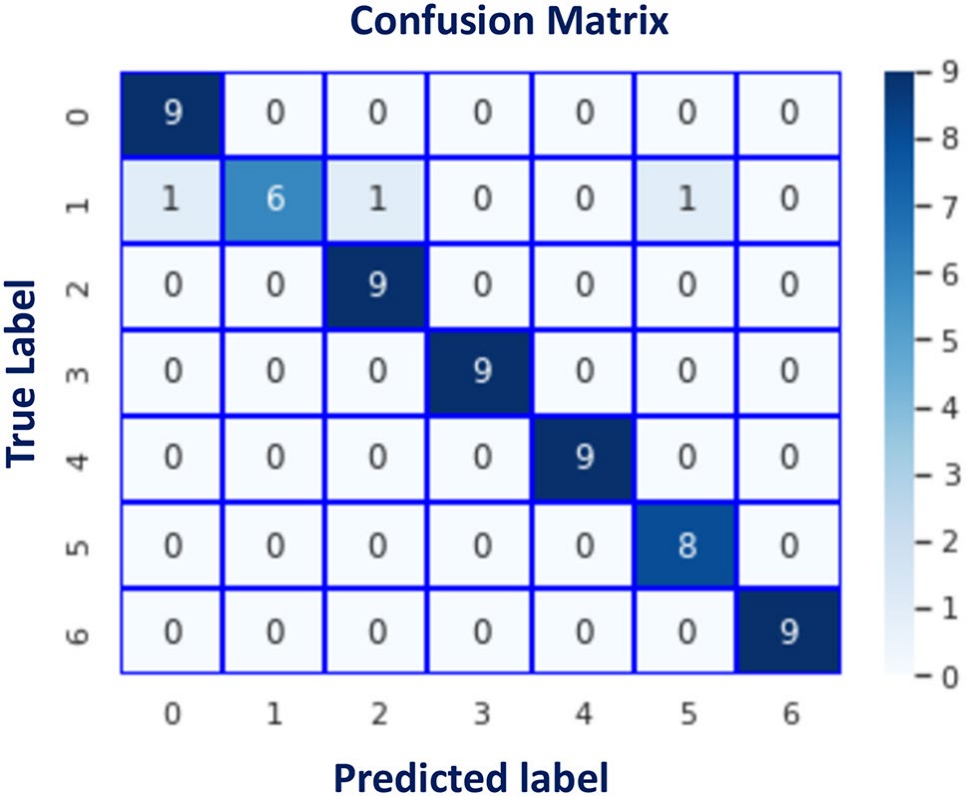
Confusion matrix using (Jaffee).

The following results have been evaluated on the Jaffee data set that was described previously in Sect. 5.1. Table 9 shows the classification-report of the Proposed deep CNN model using Jaffee dataset. Figures 9 and 10 show the accuracy, loss curves, and confusion matrix, respectively.

It is concluded that the suggested DCN model employing the Jaffee dataset has performed efficiently, with the recognition rate raising to 95% and the training accuracy reaching 95.75% in only 6 min, achieving optimal results compared to the other classical CNN models.

**Table 9:** Proposed DCN using (Jaffee).

Label	Emotion	Proposed DCN		
		Precision	Recall	F1-score
0	Anger	1.00	0.94	0.97
1	Disgust	0.89	0.87	0.88
2	Fear	0.87	0.97	0.92
3	Happy	1.00	1.00	0.00
4	Neutral	0.94	1.00	0.97
5	Sadness	0.98	0.91	0.94
6	Surprise	1.00	0.97	0.98
**Over all accuracy**				**0.95**

#### Using the RAF-DB database

**Fig. 11:**
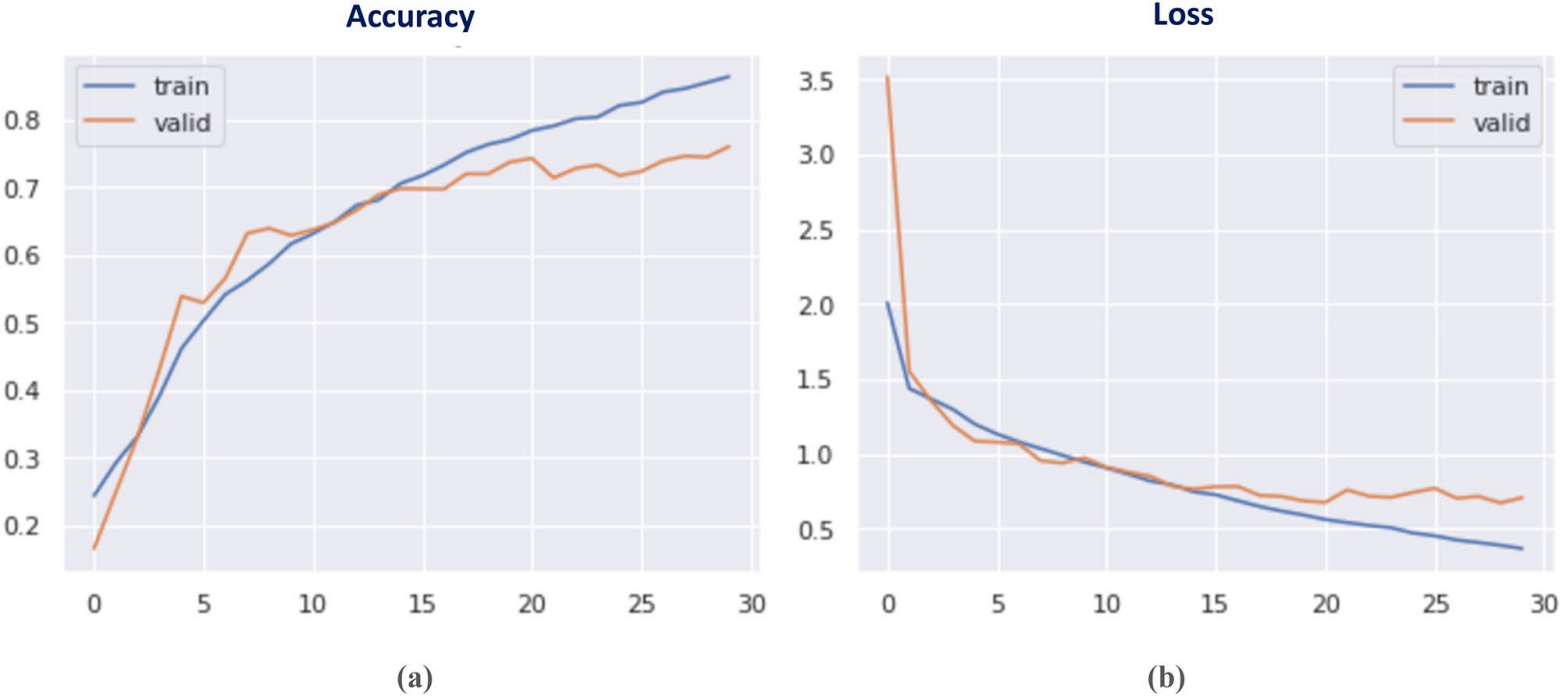
(a) Accuracy curve (b) Loss curve of RAF-DB dataset.

**Fig. 12:**
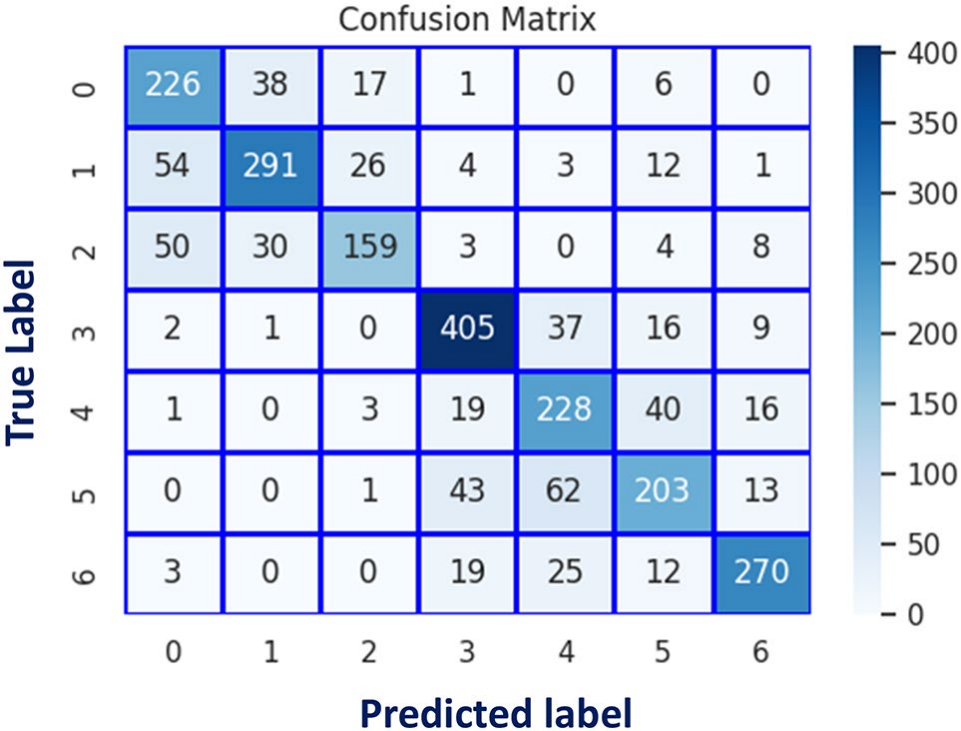
Confusion matrix using (RAF-DB).

To further understand how well the proposed model performs, another data set has been investigated. The same augmentation steps have been attempted to resolve similarity issues, but the results didn’t significantly change. Therefore, the initial data has been proceeded. Table 10 shows the proposed deep CNN using the RAF dataset. Figure 11 illustrates the accuracy and loss curves, whereas Fig. 12 provides the confusion matrix. The developed FER model, utilizing the RAF dataset, has accomplished an accuracy of 76% and a training accuracy of 93.5% in 10 min.

The authors believe that the numerous issues within the dataset that has been described in Sect. 5.1 and displayed in Table 3 may be the cause of the inadequate recognition accuracy in RAF-DB. In order to enhance the outcomes, another FER model (AA-DCN) will be introduced in this paper.

**Table 10:** Proposed DCN using RAF-DB dataset.

Label	Emotion	Proposed DCN FER		
		Precision	Recall	F1-score
0	Angry	0.64	0.88	0.74
1	Disgust	0.80	0.67	0.78
2	Fear	0.93	0.56	0.69
3	Happy	0.89	0.82	0.85
4	Neutral	0.62	0.78	0.69
5	Sadness	0.65	0.58	0.62
6	Surprise	0.83	0.87	0.85
**Over all accuracy**				**0.76**

**Fig. 13:**
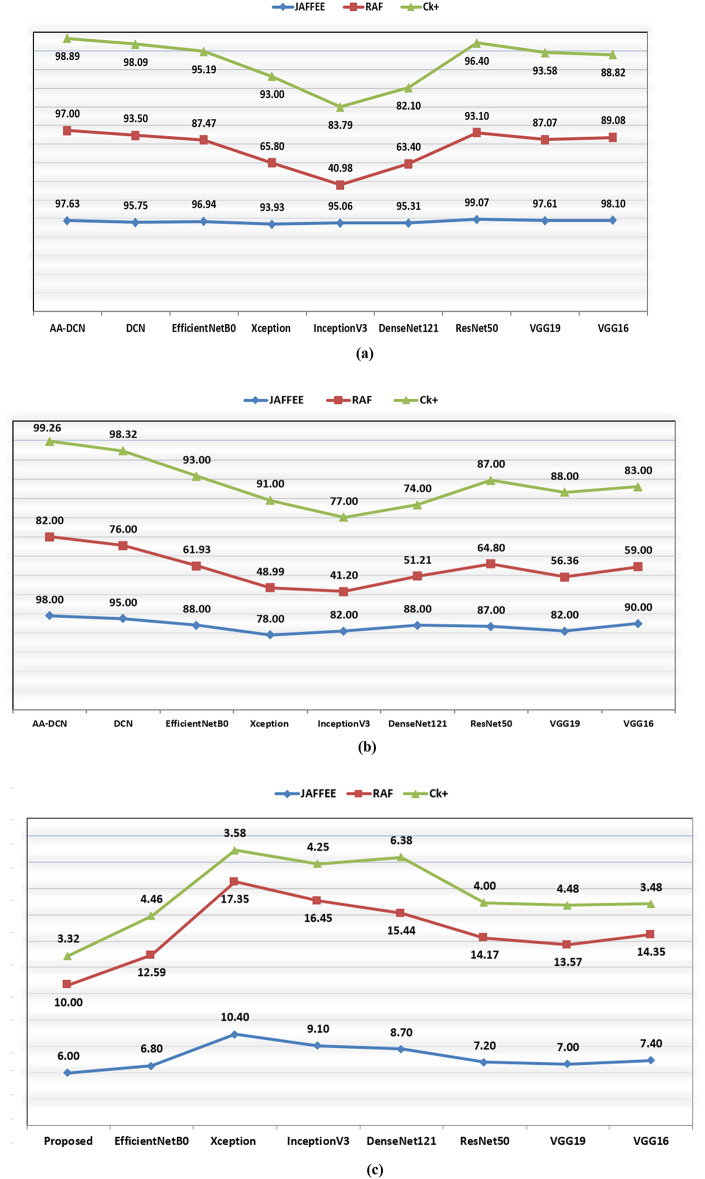
(a) Training Accuracy curve (b) Test Accuracy curve (c) Time curve for the three datasets.

Figure 13 will compare the training accuracy (a), test accuracy (b), and processing time(C) of the suggested approach which are applied to three different datasets (CK+, Jaffee, and RAF-DB) with 7-classical CNN models. Moreover, the evaluation of the classical CNN model on the three datasets is displayed in Table 11. It can be demonstrated from the results that the suggested DCN for FER methodology had delivered the most effective test and training recognition performance and required a minimum processing time. Therefore, the proposed DCN approach is computationally efficient when compared with classical CNN models.

**Table 11:** Accuracy comparison of classical CNN models applied to the three datasets.

	CK+		JAFFEE		RAF-DB	
	Test Acc	Train time	Test Acc	Train time	Test Acc	Train time
DenseNet121	74%	6.38	88%	8.7	51.21%	15.44
InceptionV3	77%	4.25	82%	9.1	41.2%	16.45
VGG16	83%	3.48	90%	7.4	59%	14.35
VGG19	88%	4.48	82%	7	56,36%	13.57
ResNet50	87%	4	87%	7.2	64.8%	14.17
Xception	91%	3.58	78%	10.4	48.99%	17.35
EfficientNetB0	93%	4.46	88%	6.8	61.93%	12.59

## Anti-aliasing and deep CNNs

Modern deep CNN models were previously believed to be invariant to tiny image alterations, but many authors have recently demonstrated that they actually are not. Their convolutional structures are a potential reason for this. Recently, DCNs have been developed with an architecture that is essentially the same: convolution and sub-sampling processes alternate. The root cause of the invariant loss is striding, while convolutions and pooling layers are in fact shift-invariant. Striding, or the subsampling process, is an aspect of both convolution and the pooling layers, and that’s where the issue arises since they usually overlook the traditional Nyquist sampling theorem.

According to that theorem, data loss (aliasing) occurs if the rate of sampling is not at least two times the highest frequency of the signal, which can have a detrimental impact on the DCN’s overall performance.

These pooling and stride convolution (down-sampling) operations provide a spatial resolution reduction of intermediate feature maps and an efficient computation.

In signal processing, “anti-aliasing” is a common technique that involves low-pass filtering (blur filtering) the input signal before sub-sampling. But the performance is decreased by merely adding this part to deep networks. Due to this, downsampling processes used in modern CNNs frequently lack anti-aliasing.

In the recent work of (Zhang, 2019)^5^, promoted an impressive architectural change in how to use this idea in today’s CNNs. The author has demonstrated that using deep CNNs with blur pooling outperforms using deep CNNs without blur filtering in terms of precision and shift-invariance when applied on the CIFAR10 dataset^35^.

These modifications are shown in the following Eqs. (13), (14), (15), and (16)^5^.


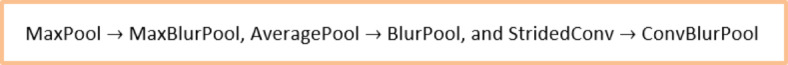

13 $$Max{\text{\_}Pool}_{{{\text{k,s}}}} \to \:\:{{Sub}\text{sample}}_{{\text{s}}} \:^{^\circ } \:\:{{Max}}_{{\text{k}}}$$

14 $$\begin{aligned} Max\_{{Pool}}_{{{\text{k,s}}}} \to & \:\:{{Sub}\text{sample}}_{{\text{s}}} \:^{^\circ } \:\:{{Blur}}_{{\text{m}}} \:^{^\circ } \:\:\:{{Max}}_{{\text{k}}} \\ = & \:\:\:BlurPool_{{m,s}} \:\:^{^\circ } \:\:\:Max_{k} \\ \end{aligned}$$

15 $$ReLU\:^{^\circ } \:\:\:Conv_{{k,s}} \to \:BlurPool_{{m,s}} \:\:^{^\circ } \:\:ReLU\:^{^\circ } \:\:\:Conv_{{k,1}} \:$$

16 $$Avg{\text{\_}}Pool_{{{\text{k,s}}}} \to \:BlurPool_{{{\text{m,s}}}}$$

where $\:{Blur}_{m}$ is an anti-aliasing filter with kernel size ($\:m\times\:m)$, and pool kernel size is donated by $\:k$, and with $\:s$ stride.

**Fig. 14:**
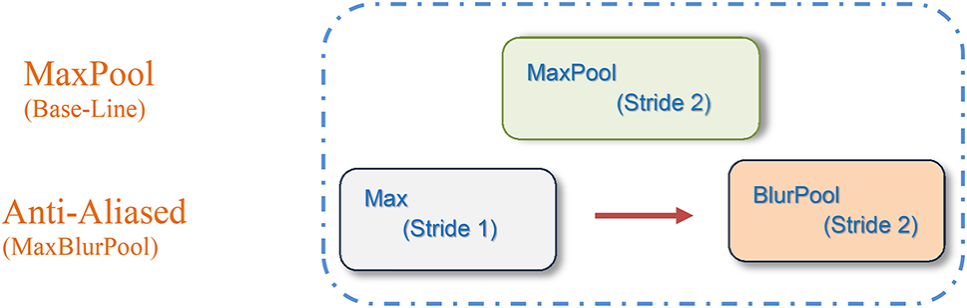
Anti-Aliased MaxPool layer.

**Fig. 15:**
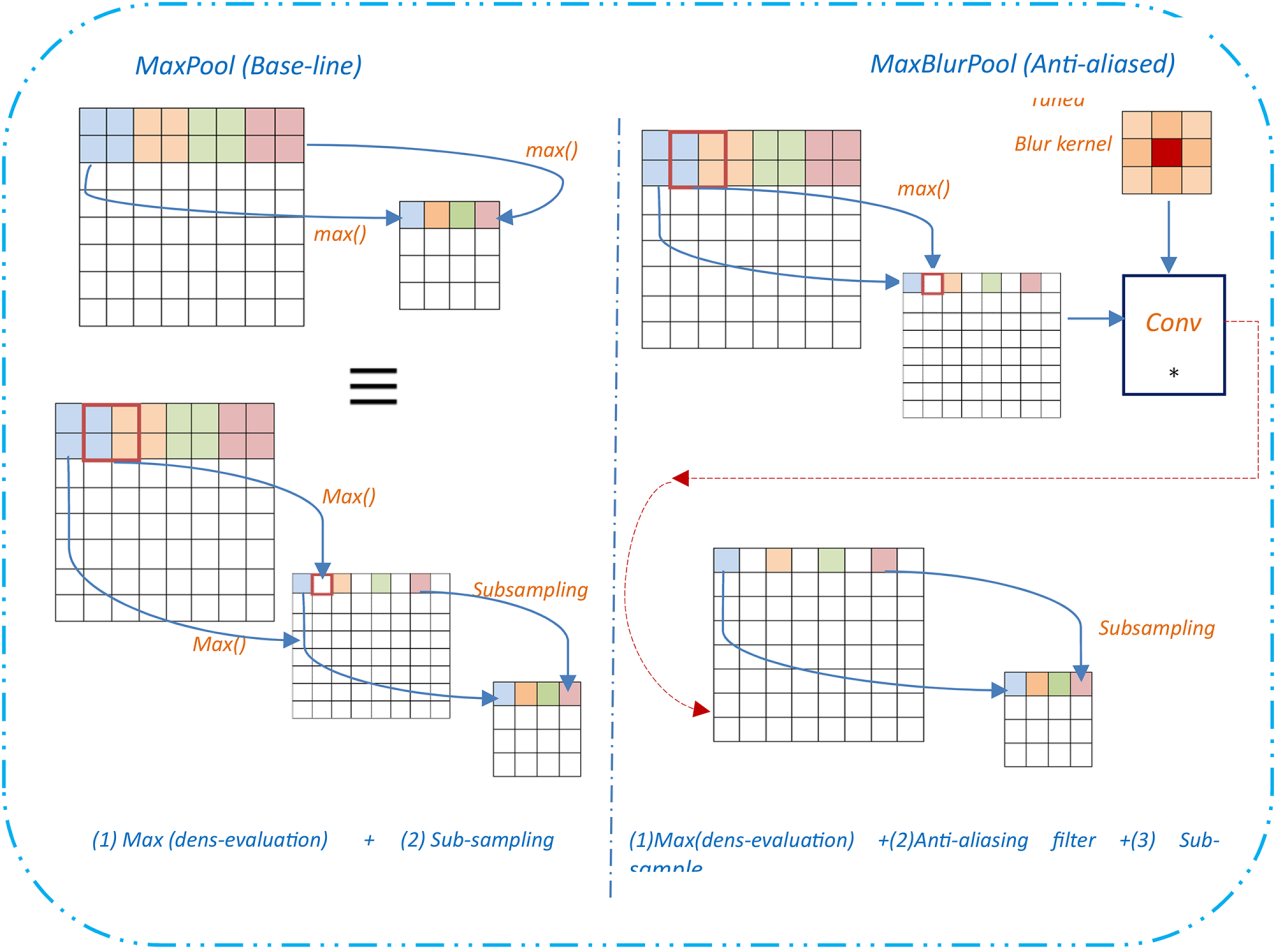
Details of MaxPool and MaxBlurPool operation.

The MaxBlurPool layer is a combination of: (i) a MaxPool layer (stride 1) that preserves shift-equivariance but no downsampling, and (ii) a BlurPool filter (stride 2), which is a combination between an anti-aliasing filter, denoted as, and subsampling. So, the blur filter has been used mainly to avoid aliasing artifacts. An illustration of the MaxBlurPooling layer is shown in Fig. 14, and Fig. 15 details the operation.

Inspired by that work, this manuscript will employ the MaxPool improvements shown in Eqs. (13), (14) in the introduced DCN model (Algorithm-1), hopefully after blurring to clear aliasing effects in the intermediate features map. This could be a promising way to be utilized for improving the emotion recognition efficiency in FER systems and specifically can increase the efficiency of challenging datasets like RAF-DB, and then it may be considered in another DCN model.

Compared to other anti-aliasing techniques for CNNs, using blur filters (like Gaussian blur) provides a strong balance of simplicity, customization, and effectiveness. Some techniques, like learned downsampling, can adapt better optimally to data, while frequency space filtering more directly minimizes aliases, and multi-scale training creates invariance to resolution changes. So, while other solutions can provide further benefits, blur filtering gives the best blend of conceptual simplicity with customizable integration while effectively improving model robustness. The intuitive nature and straightforward implementation keep it accessible and adaptable compared to more complex options.

### Algorithm 2: proposed AA-DCN overview

A novel hybrid model between antialiasing (blur filters) and the proposed DCN model, known as AA-DCN, has been deployed and applied on the three augmented CK+, JAFFEE, and RAF datasets to investigate whether applying the blur filters to anti-alias the DCNs can increase or decrease accuracy in the case of facial emotion recognition?!

As frequent reason is down-sampling (stride) strategies that disregard the sampling theorem, the proposed DCN model the has adopted stride = 1 in all conv layers, but stride = 2 with the MaxPool layers, which could be a reason to appear aliasing artifacts leads to degradation in facial emotion recognition. For this the MaxPool layers has been replaced in the proposed DCN model with the MaxBlurPool layer in the proposed AA-DCN model.

The AA-DCN model will go through the same layering structure as the proposed DCN in Sect. 4, along with alternating each MaxPool (stride 2) layer with the Max-Blur Pooling operation. Simply put, after Stage-1, a MaxPool layer (stride-1) with a kernel size of 4 × 4 and a BlurPool layer with a kernel size of 2 × 2 have been utilized. Following is Stage-2 with both MaxPool and BlurPool layers, and stage-3 followed by the same two steps. Some m × m filters ranging from sizes 2 to 5 have all been tested in the kernel tuning phase. The weights are normalized. The filters are the outer product of the following tuned vectors with themselves.


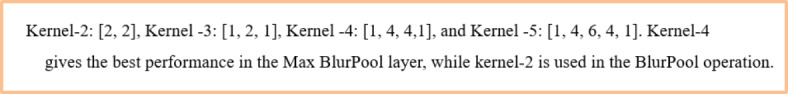


**Fig. 16:**
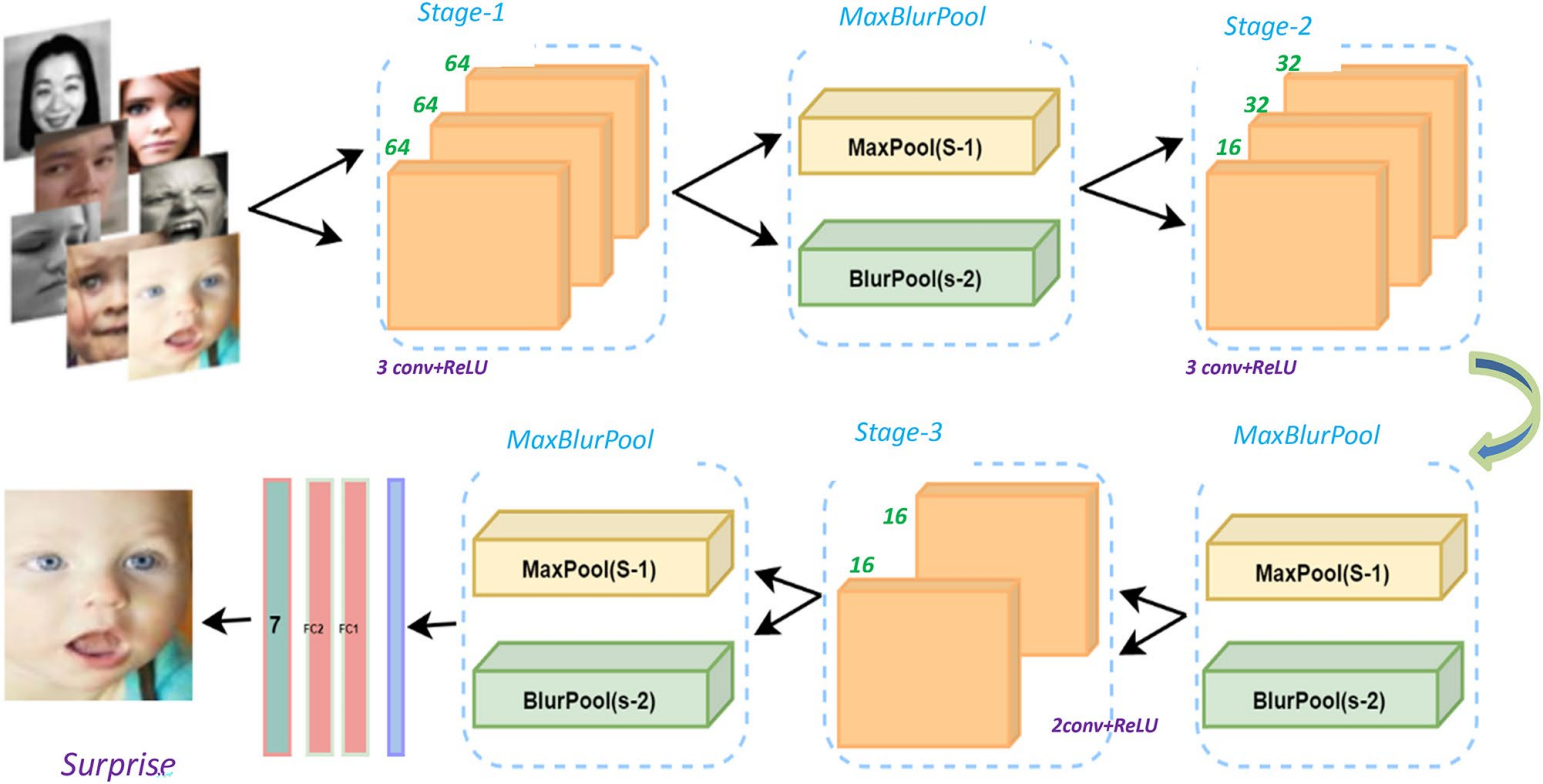
Architecture of the proposed AA-DCN.

However; Max-pool layer with kernel size of 4 × 4 and BlurPool layer with kernel size of 2 × 2 have been utilized based on the best empirical evaluation. Afterward, the flatten and the same dense layers as the proposed DCN are deployed. Also, for optimizing the hyperparameters, the Adam, Adadelta, and SGD optimizers have all been used in the tunning phase; however, SGD delivers the best results, hence it has been utilized. Figure 16 will present the overview structure of the proposed AA-DCN model.

**Table 12:** The tunned hyper-Parameter**s**.

Parameters	Value
Epochs	75
Batch size	32
Learning rate	6 × 10^**− 2**^
Momentum	0.9
Optimizer	SGD
Loss function	Spars_ categorical

Algorithm-2 illustrates the applied procedures of the MaxBlurPool layers applied on the AA-DCN model.

**Algorithm 2:**
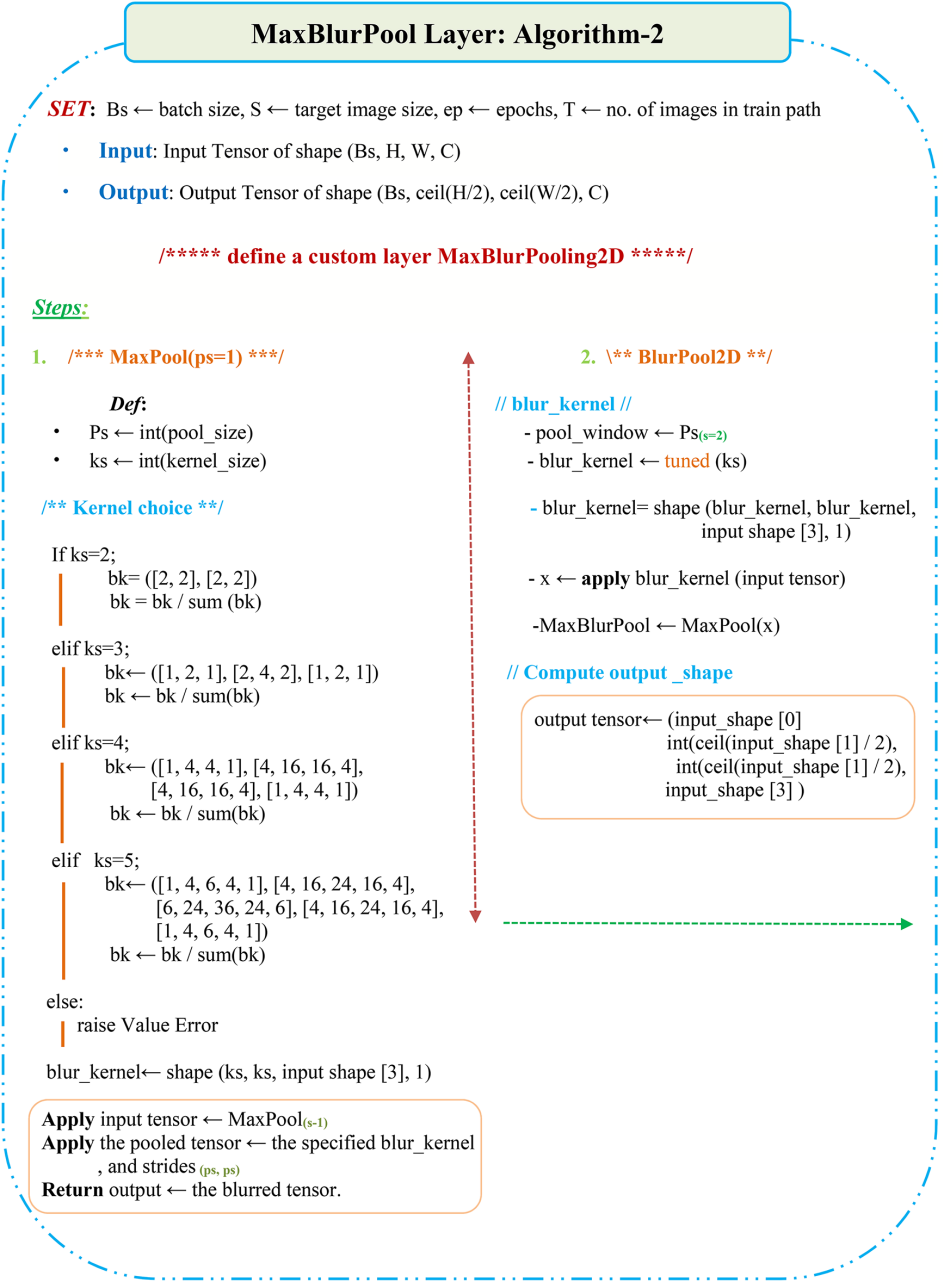
MaxBlurPool Layer.

### Results and discussion

The optimum tuning hyper-parameters in Table 12 have been used to evaluate the proposed AA-DCN model, and the same metrics in Sect. 5.3 have been utilized. Then, in the next section, the effect of adding the anti-aliased MaxPool layers on the three (CK+, JAFFEE, and RAF) datasets will be tested and the results will be compared with the suggested DCN model using the regular MaxPool layer.

#### The evaluation of AA- DCN model

The three datasets (CK+, JAFFEE, and RAF) have been used for evaluating the anti-aliased DCN model (Algorithm-2).

**Fig. 17:**
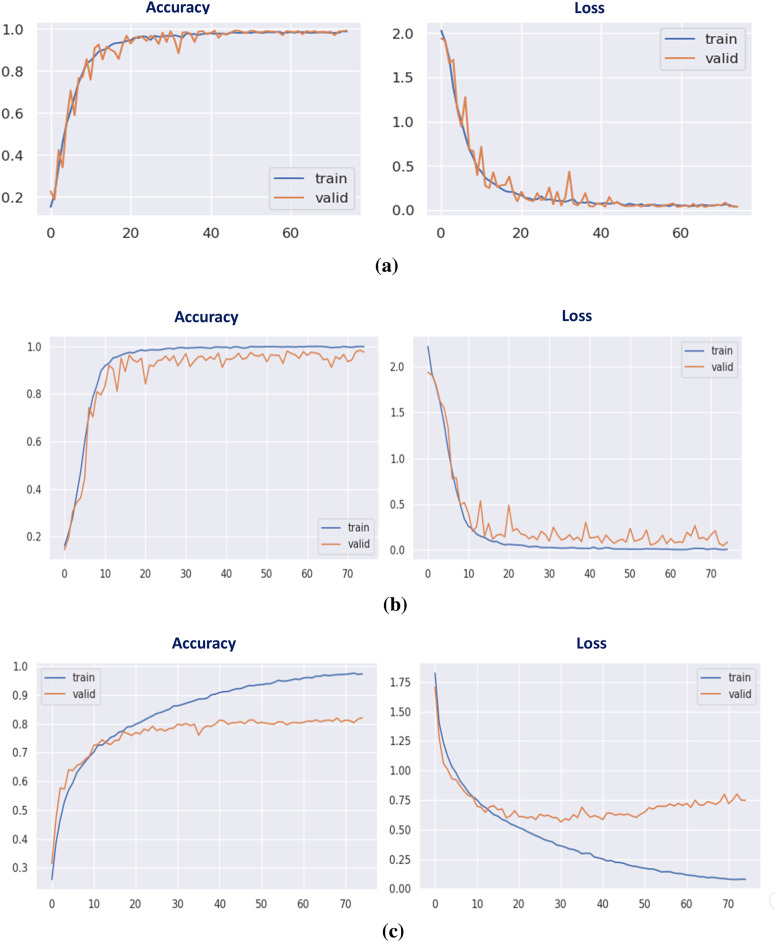
Accuracy and loss curves of the proposed AA-DCN model.

**Fig. 18:**
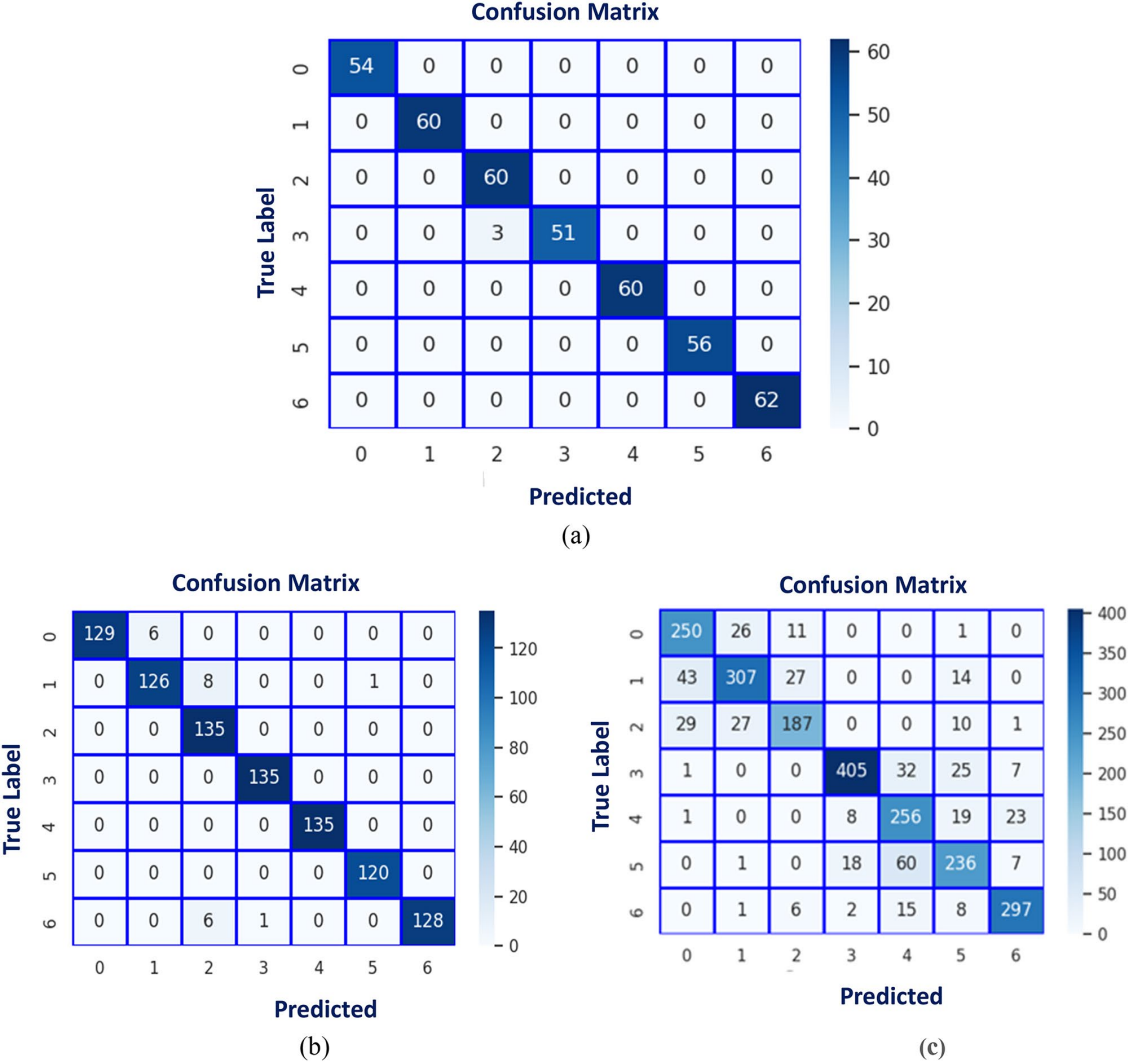
Confusion matrix of the proposed AA-DCN model applied on the three datasets.

In Fig. 17, the accuracy and loss curves are depicted. It is concluded that the suggested AA-DCN model employing the CK + dataset has performed efficiently, with the recognition rate raising to 99.26% and the training accuracy reaching 98.89% in only 3 min, 23s. Also, when employing the AA-DCN model on Jaffee dataset, it achieves a higher recognition rate of 98% and with training accuracy reaching 97.63% in only 6 min, 5s. On the RAF dataset, the AA-DCN model has 82% recognition rate with training accuracy reaching 97% in only 12 min, 2s. The confusion matrix shown in Fig. 18 has been used to derive the experimental results for the suggested model, where (a) the confusion matrix of the proposed model when applied on CK + dataset, (b) using Jaffee, and (C) when utilizing the RAF dataset. Table (13), (14), and (15) illustrate the classification reports of the proposed AA-DCN model applied to the CK+, Jaffee, and RAF datasets, respectively. Moreover, each table illustrates the precision, recall, and F1-score results for each emotion and also the overall accuracy of the proposed model for each utilized dataset.

**Fig. 19:**
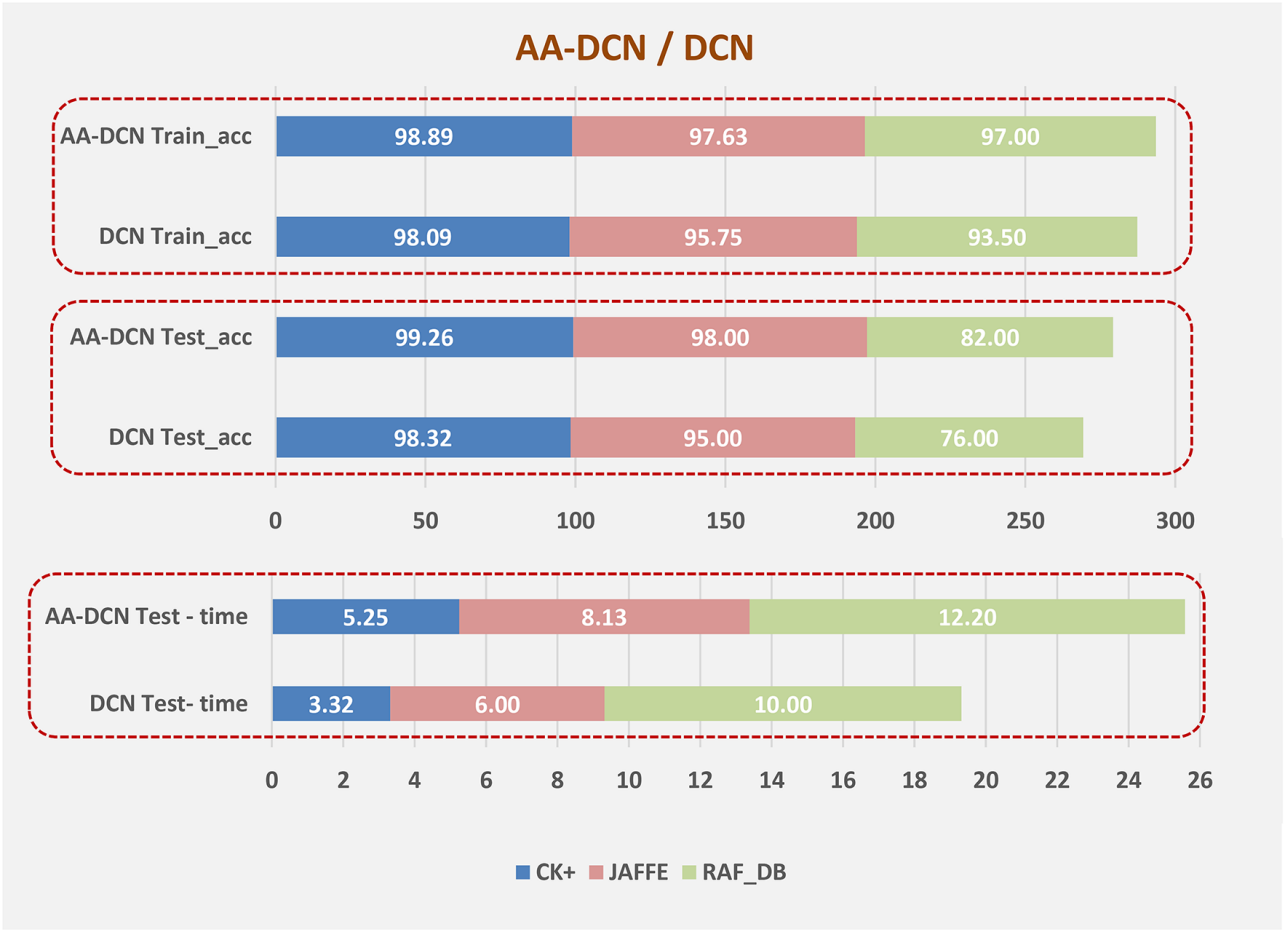
Comparison between the two suggested models.

Table 16; Fig. 19 show a comparison between the evaluated results from the DCN (Algorithm-1) and AA-DCN (Algorithm-2) models that have been conducted on the three datasets. The results of this study demonstrate that it is possible to considerably increase the invariance of networks trained on face emotion datasets by altering the two layers of the MaxBlurPool operation instead of the conventional Max-Pool layers and then retraining. The proposed AA-DCN methodology has scored 99.26% in the case of using dataset CK + in 5.25 m; moreover, it scored 98% in the JAFFEE dataset in 8.13 m, which improved the accuracy value by 3% despite using more epochs—from 30 to 75 than Algorithm-1, which applied the natural MaxPool layers. In the case of the Raf-DB, the anti-aliased methodology (Algorithm-2) outperforms the results from Algorithm-1 by 6% in 12.2 m.

**Table 13:** Proposed AA-DCN using Ck +.

Label	Emotion	DCN	Proposed FER		
		Precision	Recall		F1-score
0	Anger	1.00	1.00		1.00
1	Contempt	1.00	1.00		1.00
2	Disgust	0.95	1.00		0.98
3	Fear	1.00	0.94		0.97
4	Happy	1.00	1.00		1.00
5	Sadness	1.00	1.00		1.00
6	Surprise	1.00	1.00		1.00
**Over all accuracy**					**0.99**

**Table 14:** Proposed AA-DCN Jaffee.

Label	Emotion	Proposed AA-DCN FER		
		Precision	Recall	F1-score
0	Angry	1.00	0.96	0.98
1	Disgust	0.95	0.93	0.94
2	Fear	0.91	1.00	0.95
3	Happy	0.99	1.00	1.00
4	Neutral	1.00	1.00	1.00
5	Sadness	0.99	1.00	1.00
6	Surprise	1.00	0.95	0.97
**Over all accuracy**				**0.98**

**Table 15:** Proposed AA-DCN using RAF dataset.

Label	Emotion	Proposed AA-DCN FER		
		Precision	Recall	F1-score
**0**	Angry	0.77	0.87	0.82
**1**	Disgust	0.85	0.79	0.82
**2**	Fear	0.81	0.74	0.77
**3**	Happy	0.94	0.86	0.90
**4**	Neutral	0.71	0.83	0.76
**5**	Sadness	0.75	0.73	0.74
**6**	Surprise	0.89	0.90	0.89
**Over all accuracy**				**0.82**

**Table 16:** Summary of the evaluation outcomes for the two suggested models.

Dataset	Proposed DCN (30 epochs)				Proposed AA-DCN (75 epochs)			
	Train_acc (%)	Test_acc (%)	Avg epoch_ time(s)	Train- time (m)	Train_acc (%)	Test_acc (%)	Avg-epoch_ time(s)	Train - time (m)
**CK+**	98.09	98.32	6.64	3.32	98.89	99.26	4.2	5.25
**JAFFE**	95.75	95	12	6	97.63	0.98	6.5	8.13
**RAF_DB**	93.5	76	18	10	97	0.82	9.7	12.2

To demonstrate that the proposed AA-DCN with the MaxBlurPool layers provides the best level of precision compared to other pooling techniques, this work evaluates this model on the three datasets further, but with the MaxBlurPool layers replaced with average pool layers as an additional analysis that reveals which of these pooling techniques has the most prevalent FER reliability. Table 17 compares MaxBlurPool layers based on the AA-DCN model to Average Pool layers based on the AA-DCN technique.

**Table 17:** MaxBlurPool layers and Average Pool layers applied to the three datasets.

	MaxBlurPooling Acc.	Average Poling Acc.
CK+	99.26%	98.03%
JAFFEE	98%	91.13%
RAF-DB	82%	79.54%

**Table 18:** ANOVA test for FER on AA-DCN algorithm.

Datasets		SS	DF	MS	F-value	*P*-value
CK+	Between groups Within groups Total	SST: 0.0003 SSE: 0.0028 SSR: 0.16	df_groups = 6 df_error = 400 df_total = 406	6.8796e-06	1.013	0.42 (*p* < 0.05)
Jaffee	Between groups Within groups Total	SST: 0.372 SSE: 0.0149 SSR: 0.357	df_groups = 6 df_error = 923 df_total = 929	0.16022	8.146	1.34e-08 (*p* < 0.00000001)
RAF	Between groups Within groups Total	SST: 0.8965 SSE: 0.1298 SSR: 0.76674	df_groups = 6 df_error = 2354 df_total = 2360	0.5498	3.014	0.0062 (*p* < 0.05)

**Table 19:** Wilcoxon test for FER on AA-DCN algorithm.

	CK+	Jaffee	RAF
Number of values	5	18	495
Wilcoxon signed rank test			
Sum of signed ranks (W)	2	9	332
Sum of positive ranks	3	57	465
Sum of negative ranks	2	9	332
Exact or estimate?	Exact	Exact	Exact
P value summary	***	***	***
Significant (alpha = 0.01)?	Yes	Yes	Yes
How big is the discrepancy? Discrepancy	1	48	133

**Table 20:** Unsolved challenges and a proposed design of solution.

	Unsolved challenges	Solution in the proposed model
1	Limitation no. of samples in CK+, Jaffee datasets.	Augmentation to increase no. of samples for high accurate training.
2	Unbalance samples in emotion folders in RAF dataset.	Augmentation to balance no. of samples in each emotion for efficient and accurate classification.
3	Poor quality and different real-world scenarios (occlusion) existing in RAF dataset	Proposing AA-DCN model to enhance the classification task of RAF, a real word dataset, (one of the most challenging dataset).
4	The anti-aliasing problem that appears due to down sampling in traditional CNN models, that leads to miss classification tasks	With challenging datasets like RAF, the suggested AA-DCN model successfully overcomes the anti-aliasing phenomena leading to a significant increase in FER task.

**Table 21:** Comparing the overall performance with other studies.

Overall performance comparison employing CK + datasets with various methods		
Reference/Year		Accuracy
Fontaine et al. /2022^21^		89.7%
Abate et al. /2022^10^		90.42%
Lu et al. /2022^23^		95%
Pusarla et al. /2022^33^		95.21%
Khattak et al./2021^12^		95.65%
Chowdary et al./ 2021^9^		96%
Shaik et al. /2022^11^		97.67%
Umer et al./ 2021^3^		97.69%
Helay et al./ 2023^35^		98%
Debnath et al./ 2022^22^		98.13%
Proposed DCN		**98.32%**
roposed AA-DCN		**99.26%**

Consequently, the main contribution of this research, is to provide an answer to the question posed in Sect. 7.1, “Will applying the blur filters to anti-alias the DCNs increase or decrease accuracy in the case of facial emotion recognition?“. The answer is obviously “yes,” as anti-aliased MaxPooling enhances intermediate feature maps, which in turn significantly enhances recognition accuracy, particularly with tricky data like RAF. In the proposed AA-DCN model, the right selection of the layering structure with the fine-tuned hyperparameters is a significant step to get high recognition rate in minimum time, less complexity of the proposed model is another factor helps in enhancing the recognition rate, but adopting the MaxBlurPool instead of MaxPool layers in the proposed model plays a crucial rule to get the best recognition rate.

Additionally, Table 18 displays the test results of the proposed model using a one-way ANOVA test. Table 19, on the other hand, presents the results of a Wilcoxon Signed-Rank test model comparison. The table observations indicate that there is a substantial variation in the model’s performance on distinct emotion classes, with the p-value scoring below 0.05 even when applied to three different datasets. This is consistent with predicted behavior since emotions naturally vary in complexity and facial expressions. Also, it shows that the model has adapted to the emotion-specific patterns and differences in the dataset, rather than treating all emotions as the same. Be aware that a model that functions flawlessly across all emotions can be overgeneralized and miss crucial details.

Some of the unsolved challenges and the solution offered by the proposed model is shown in Table 20. Accordingly, a comparative study has been formed with recent researches in facial expression recognition. This comparison is introduced in Table 21, which shows that the best performance was around 98% in the original CK + dataset, which has a small number of sample images. However, the recommended methodology; has targeted about 98.32% with an augmented larger-sized CK + dataset in case of applying algorithm-1 and 99.26 in case of applying the anti-aliasing model (AA-DCN). In comparison to models with seven expressions, the proposed AA-DCN-based model is more accurate by around (1–8) %.

Generally, the most noteworthy outcomes from this research are:

- The current FER model topology outperforms the prior architecture of standard CNN models in terms of accuracy.
- The claimed DCN technique balances processing time and efficiency adequately; however, adding a Max Blur Pool layer greatly increases the rate of emotion identification, and it should be adapted for implementation with different network topologies to enhance face expression recognition performance.
- Even though they may require a bit more recognition time due to incorporating a further stage (a blur filter), the anti-aliasing layers can detect emotion much more effectively and precisely.

To keep things simple, CNNs can effectively learn hierarchical patterns in facial features (edges, textures, and shapes). Facial images have a spatial structure well-suited for convolutional feature learning. However, faces contain textures that can alias without antialiasing. This can hurt fine-grained expression classification. Expression changes can be very subtle, so anti-aliasing improves signal clarity. It improves model robustness to image variations, orientations, etc. Overall, CNNs align well architecturally to extract visual features from faces. Antialiasing then makes the model more robust to aliasing-based degradation of those facial cues, which could worsen performance for subtle expression recognition. The combination enables reliably discernible facial feature extraction and representation learning for sensitive expression classification tasks. So they complement each other nicely for this application area.

## Conclusion and future scope

In this paper, two innovative FER models based on deep CNN have been introduced. Initially, a DCN model has been designed and improved with the traditional MaxPool layers and by fine adapting its parameters to analyze facial expressions. For emotion categorization, a group of eight different and distinctive emotion classes has been focused on. The experiments conducted on three publicly available datasets illustrate how convolutional networks with strides can be demonstrated and their impact on the emotion recognition issue.

The proposed DCN methodology’s validation accuracy has reached 98.32% and 95% as a result of the Ck + and Jaffee datasets, respectively, and an average accuracy of 76% using the Raf dataset with the minimum number of epochs and the lowest processing time compared with classical models. The average result from the Raf data set demonstrates that similar to face biometric recognition, occluded faces continue to be a challenging limitation for computer vision solutions. However, this paper provides a solution: by implementing the AA-DCN model on the RAF dataset, the recognition rate has been significantly increased by 6%, showing the effect of anti-aliasing in improving the FER accuracy.

The second model (AA-DCN) has been subsequently developed by altering the MaxPool layer with the anti-aliased MaxPool processes. The AA-DCN has also been used on the same data sets to study and investigate the influence of applying a tuned low-pass filter prior to subsampling operations in the MaxPool layer, as well as its collateral effects on recognizing emotions in FER models. For the Ck + and Jaffee datasets, the AA-DCN technique has achieved 99.26% and 98%, respectively. Additionally, a comparison study between the suggested model and other recent studies has been conducted. Both of the suggested models outperform other current methods, according to experimental results.

In conclusion, incorporating blur filters is a promising way to enhance accuracy and ought to be taken into consideration when developing current deep CNNs. For future work, a plan to apply the anti-aliasing process to complex types or mixed groups of emotions has been taken into consideration, such as being shocked by pleasure, surprised by frustration, dissatisfied by anger, surprised by sorrow, and so on.

## Data Availability

The data that support the findings of this study are available within this article.
